# Microbial-Responsive Wound Dressings Based on Biopolymer Degradation Strategy for Detecting Bacterial Infections

**DOI:** 10.1021/acsami.5c22357

**Published:** 2026-01-27

**Authors:** Sara Sadati, Marcus J. Swann, Steven L. Percival, Jerome Charmet, Meera Unnikrishnan, Dmitry Isakov

**Affiliations:** † Division of Biomedical Sciences, Warwick Medical School, 2707 University of Warwick, Coventry CV4 7AL, United Kingdom; ‡ WMG, University of Warwick, Coventry CV4 7AL, United Kingdom; § 5D Health Protection Group, Accelerator Building, 1 Daulby Street, Liverpool L7 8XZ, United Kingdom; ∥ School of Biomedical and Precision Engineering, University of Bern, Bern 3008, Switzerland; ⊥ School of Engineering HE-Arc Ingénierie, HES-SO University of Applied Sciences and Art of Western Switzerland, Neuchâtel 2000, Switzerland

**Keywords:** enzyme-responsive materials, bacterial infection detection, physical sensors, wound monitoring, quartz crystal microbalance, smart wound dressings

## Abstract

Chronic wounds remain a major clinical challenge due to their strong association with antibiotic-resistant microbial biofilms. These nonhealing wounds demand advanced therapeutic strategies that go beyond passive protection to actively monitor and respond to changes in the wound environment. To address this, we propose an activity-based sensing strategy that detects bacterial proteolytic activity using composition-tunable biopolymer films that degrade in response to pathogen-secreted enzymes. Gelatin films cross-linked with (3-glycidyloxypropyl)­trimethoxysilane (GPTMS) and blended with poly­(ethylene oxide) (PEO) were engineered to undergo selective peptide-bond cleavage by proteolytic activity. The incorporation of PEO enhanced water uptake and accelerated enzymatic degradation, with the optimal composition (25% PEO) exhibiting 4-fold faster mass loss compared to cross-linked gelatin, reaching ∼80% degradation within 12–24 h in the presence of the bacterial pathogen *Pseudomonas aeruginosa* and ∼35% within 24–48 h with drug resistant *Staphylococcus aureus*. Real-time acoustic measurements revealed distinct degradation kinetics and viscoelastic signatures at nanoscale that correlated with *P. aeruginosa* protease activity, while Fourier-transform infrared spectroscopy and scanning electron microscopy confirmed structural and morphological changes following enzymatic exposure. Together, these findings establish a label-free, enzyme-responsive sensing platform that transduces bacterial activity, including biofilm-associated proteolysis, into quantitative physical signals. These findings establish composition-tunable enzyme-responsive biopolymer degradation as a viable broad-spectrum platform responding to total proteolytic activity. As no pathogen-specific recognition elements are required, this platform offers excellent potential to detect challenging polymicrobial infections.

## Graphical abstract

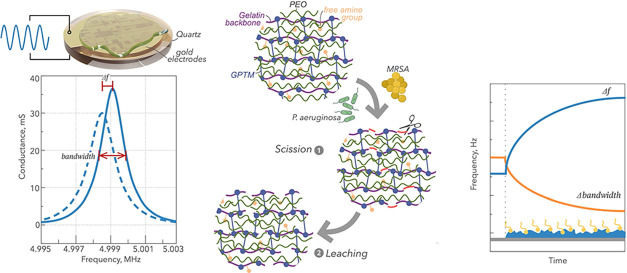

## 1 Introduction

Chronic wounds represent a major global healthcare burden, affecting millions of patients annually, incurring costs in excess of $148 billion globally.^1^ The transition from acute to chronic wound status frequently results from bacterial colonization and biofilm formation, which helps increase antimicrobial resistance, and which occurs in 60–90% of chronic wounds. Wound biofilms are associated with increased antimicrobial resistance and can severely impair healing through sustained inflammation and tissue destruction.^2–4^

Early detection of wound infection is critical for timely intervention. However, current clinical practice relies primarily on visual assessment and subjective interpretation of infection signs, methods that often fail to detect bacteria until infection is well-established.^5^ While laboratory culture-based diagnostics can provide definitive identification, they require 24–72 h to obtain results, creating significant delays between infection onset and appropriate treatment.^6,7^ Other methods, including biopsy, molecular-based assays, and microscopy, often require specialized facilities or can be costly and invasive, which limit their widespread use in the clinical workflow.^8,9^ While optical/fluorescent imaging-based methods are providing promising advances at point-of-care technologies,^10^ current advice is often to treat wounds according to the risk of infection or under the assumption of the presence of biofilms for chronic wounds.^11,12^ This diagnostic gap highlights an urgent need for point-of-care wound infection sensors capable of detecting bacterial presence rapidly, reliably, and without requiring complex laboratory infrastructure.^13^

Emerging smart wound dressings have sought to address this need by integrating sensing functionality directly into the dressing material, enabling continuous monitoring of wound biomarkers.^14^ Most common approaches detect pH changes,^15^ temperature variations,^16^ or biomarkers such as uric acid^17^ and glucose^18^ that indicate general wound status. More recently, these biomarkers are implemented through stimuli-responsive polymeric materials, where variations in pH or temperature induce swelling, deswelling, or solubilization that can trigger drug release or antimicrobial activity.^19–22^ However, changes in these markers can also be caused by a variety of other factors, therefore lacking specificity for bacterial infection and biofilm formation.^23^ More advanced systems target species-specific markers such as pyocyanin from *Pseudomonas aeruginosa* ^24,25^ or employ immunoassay-based detection,^26,27^ but these approaches face significant limitations. Single-pathogen sensors are unable to detect polymicrobial infections which are common in most chronic wound infections, where multiple bacterial species contribute to pathology.^25^ Immunoassay-based systems often require complex synthetic receptors or antibodies that increase costs, reduce shelf stability, and provide binary readouts without information about infection severity or progression.^28^ Importantly, many existing platforms focus on detecting bacterial presence rather than bacterial activity, potentially generating false signals from dead bacteria or nonviable contamination and missing early stage infections where bacterial numbers are low but metabolic activity is high.^29^

Here we explore a fundamentally different approach based on detecting bacterial proteolytic activity rather than bacterial presence or static biomarkers. Common wound associated pathogenic bacteria including *P. aeruginosa* and *Staphylococcus aureus* secrete extracellular proteases as key virulence factors that facilitate tissue invasion, nutrient acquisition, and immune evasion.^30–33^ Importantly, protease secretion is closely linked to bacterial metabolism and virulence, making proteolytic activity a functional biomarker that reflects both infection onset and severity.^33^ Enzyme-responsive biomaterials that exploit pathogen-secreted enzymes have been reported previously to induce material degradation or trigger antimicrobial release, through cleavable natural polymers or engineered synthetic scaffolds designed to respond within biofilms.^34–36^

Despite demonstrating the promise of targeting bacterial enzymatic activity, these studies mostly rely on optical readouts such as colorimetric or fluorescence changes.^37–39^ Understanding the real-time kinetics and mechanism of enzymatic film degradation is essential for rational design of infection-responsive materials, whether for triggered antimicrobial release applications or as sensing layers for practical transduction platforms. Quartz crystal microbalance (QCM) techniques provide an analytical approach for this purpose, directly transducing enzymatic film degradation into measurable frequency shifts proportional to mass changes at the nanoscale.^40,41^ Beyond simple mass detection, QCM with impedance (QCM-I) simultaneously monitors bandwidth changes that report on viscoelastic properties, enabling real-time, label-free characterization of how films transition from rigid to soft states during enzymatic activity.^42^ Previous QCM studies demonstrated protease detection using peptide-cross-linked hydrogels or protein films, achieving nanomolar sensitivity for purified enzymes such as elastase and matrix metalloproteases.^43,44^ However, these studies employed purified enzymes in simplified buffer conditions, with limited investigation of responses to complex protease mixtures secreted by live bacterial cultures under wound-relevant conditions.

In this work, we develop and characterize enzyme-responsive gelatin films cross-linked with (3-glycidyloxypropyl)­trimethoxysilane (GPTMS) and blended with poly­(ethylene) oxide (PEO) as tunable sensing layers for detecting bacterial proteolytic activity based on gelatin, a denatured collagen derivative that serves as a natural substrate for bacterial gelatinases.^45–47^ The study focuses on correlating compositional changes with degradation behavior using gravimetric and acoustic analysis. Fourier-transform infrared spectroscopy and scanning electron microscopy are used to confirm structural and morphological changes following enzymatic exposure, while QCM-I provides quantitative, real-time monitoring of mass loss and viscoelastic changes during degradation by *P. aeruginosa*. By systematically varying the polymer composition, we demonstrate how these parameters control the rate and mode of enzymatic film breakdown. Figure 1 depicts the detection approach taken in this work to enable real-time monitoring of bacterial proteolytic activity. In this work, gelatin was selected as an established, biologically relevant protease-sensitive substrate, allowing to focus on how proteolytic activity is transduced and resolved. This study provides in-depth, real-time characterization of bacterial proteolytic degradation of a biomaterial coating using QCM-I, enabling simultaneous resolution of mass loss, viscoelastic changes, and interfacial dynamics that are inaccessible using conventional end point or optical assays.

**Figure 1:**
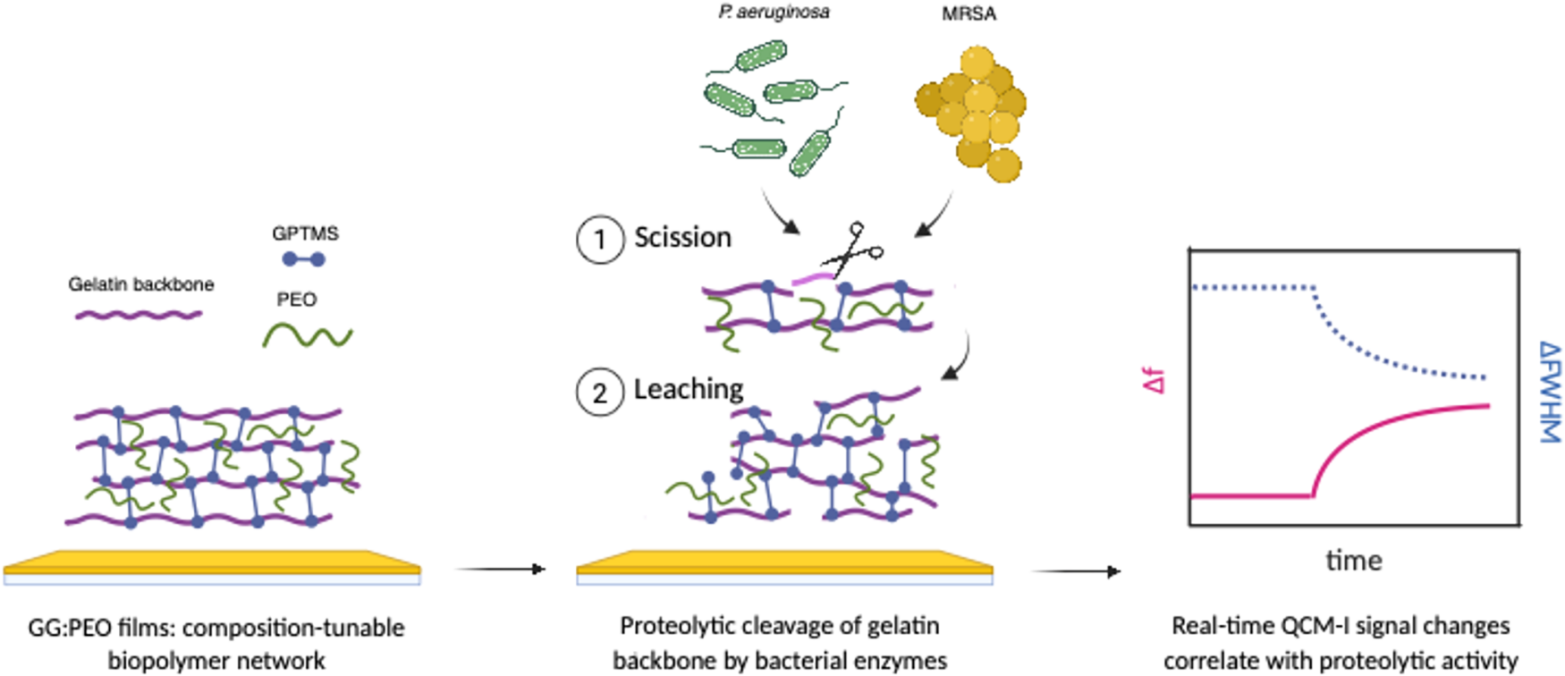
Schematic illustration of the detection approach in this work. GG:PEO films hybrid films were prepared and exposed to bacterial environments. Based on our two-step degradation model, the biopolymer network chains were cleaved and leached from the surface, leading to film degradation. The mass loss and viscoelastic changes were monitored in real-time by frequency and bandwidth changes using QCM-I.

The findings establish a material platform capable of reporting bacterial enzyme activity through measurable mass and mechanical changes, offering a foundation for future infection sensing, wound monitoring technologies, and stimuli-responsive therapeutics.

## 2 Experimental Section

### 2.1 Materials

Gelatin powder from porcine skin (type A, 300 bloom) and (3-glycidyloxypropyl)­trimethoxysilane (GPTMS) were purchased from Sigma-Aldrich (St. Louis, MO, USA). Poly­(ethylene oxide) (PEO, molecular weight 300 000 g/mol) was obtained from α Aesar (Haverhill, MA, USA). Tryptic Soy Broth (TSB) and Luria–Bertani (LB) broth were sourced from Sigma-Aldrich. Hydrogen peroxide solution (30%) was obtained from Sigma-Aldrich, and ammonia solution (32%) was supplied by VWR Chemicals (Radnor, PA, USA). Milli-Q water (18.2 MΩ·cm at 25 °C) was used for all aqueous solutions. All materials were of reagent grade and used without further purification unless otherwise stated.

Gold-coated quartz crystal sensors (14 mm diameter, 5 MHz fundamental frequency) were purchased from Semilab Co. Ltd. (Budapest, Hungary).

### 2.2 Synthesis of Gelatin-GPTMS-PEO Hybrid Films

Gelatin solutions (5% w/v) were prepared by dissolving gelatin powder in deionized water at 50 °C with stirring until fully dissolved. For cross-linked gelatin (GG) films, GPTMS was added and mixed for 1 h at 50 °C to initiate the epoxy-amine cross-linking reaction. This process follows a two-stage mechanism where GPTMS first reacts with amine groups in gelatin through epoxy ring-opening, then the trimethoxysilane groups hydrolyze and condense to form siloxane (Si–O–Si) cross-links between gelatin chains.^48,49^

Cast gelatin films were prepared using three cross-linked gelatin formulations, denoted GG1:10, GG2:10, and GG3:10, which were prepared by varying the mass ratio of GPTMS to gelatin at 1:10, 2:10, and 3:10, respectively. For each formulation, the volume of solution cast per Petri dish was adjusted such that the total dry solids were constant (0.5 g) and the resulting film thicknesses were comparable. Table 1 summarizes the calculated gelatin and GPTMS contents of each formulation, corresponding to approximately 9, 16.6, and 23 wt % GPTMS in the dried films.

**Table 1:** Gelatin and GPTMS Contents in Solvent-Cast GG Films with Varying Crosslink Density

film formulation	nominal GPTMS: gelatin mass ratio (w/w)	gelatin (w/w)	GPTMS (w/w)
GG1:10	1:10	91.0%	9.0%
GG2:10	2:10	83.4%	16.6%
GG3:10	3:10	77.0%	23.0%

For gelatin-PEO hybrid films, both gelatin and PEO stock solutions were prepared at 10% w/v concentration. The gelatin solution was prepared as described above with GPTMS addition (2:10 mass ratio), while PEO was dissolved in deionized water at 60–80 °C with gentle stirring. A molecular weight of 300,000 g/mol was selected based on preliminary studies showing that this intermediate molecular weight provided sufficient chain entanglement for film formation while maintaining adequate solubility and processability during solution blending. Blended films were then produced by mixing these stock gelatin–GPTMS (GG) and poly­(ethylene oxide) (PEO) solutions at nominal mass ratios of 100:0, 85:15, 75:25, 67:33, and 60:40 (w/w), denoted GG100 (GG2:10), GG85:PEO15, GG75:PEO25, GG67:PEO33, and GG60:PEO40, respectively. In all formulations, the GPTMS to gelatin mass ratio was fixed at 2:10. Casting volumes were adjusted so that each film contained approximately 0.5 g total solids, giving comparable thicknesses. Solutions were cast into Petri dishes and air-dried at room temperature for 48 h. Film thicknesses were measured using a digital caliper as 65 μm ± 5 μm. Table 2 summarizes the corresponding masses and weight fractions of gelatin, GPTMS, and PEO in the dried films.

**Table 2:** Gelatin, GPTMS, and PEO Contents in GG:PEO Films

film formulation	nominal GG: PEO (w/w)	gelatin (w/w)	GPTMS (w/w)	PEO (w/w)
GG100	100:0	83.4%	16.6%	0%
GG85:PEO15	85:15	71.4%	14.3%	14.3%
GG75:PEO25	75:25	62.5%	12.5%	25.0%
GG67:PEO33	67:33	55.6%	11.1%	33.3%
GG60:PEO40	60:40	50.0%	10.0%	40.0%

### 2.3 Bacterial Culture and Treatment Protocols

Bacterial inocula were prepared from glycerol stocks cultured overnight on Luria broth (LB) with agar plates for *P. aeruginosa* PA14 and tryptic soy agar (TSA) plates for methicillin resistant *S. aureus* (MRSA) JE2 strain at 37 °C. Single colonies were transferred to LB and TSB liquid media respectively and incubated overnight at 37 °C with shaking at 180 rpm until an optical density (OD_600_) of 1.0 was reached, corresponding to approximately 8 × 10^8^ CFU/mL for *P. aeruginosa* PA14 and 5 × 10^8^ CFU/mL for MRSA JE2. Working cultures were diluted to OD_600_ = 0.05 in fresh medium for film exposure experiments.

Circular film samples (2.5 cm diameter) were sterilized under UV radiation (254 nm) for 10 min and placed in individual wells of sterile 12-well plates. Control experiments confirmed that this UV sterilization protocol did not measurably affect baseline film properties, with FTIR spectra and swelling behavior remaining equivalent to nonsterilized samples stored under sterile conditions (data not shown). Bacterial suspensions were added to designated wells and films were exposed for 6, 12, or 24 h to *P. aeruginosa* PA14, and 12, 24, or 48 h to MRSA JE2 growing cultures. Control samples were incubated with sterile nutrient medium under identical conditions. Following treatment, films were thoroughly rinsed with sterile phosphate-buffered saline (PBS, 0.01 M, pH 7.4) to remove loosely attached bacteria and culture medium before characterization.

Extracellular protease activity was qualitatively assessed using 3% w/v gelatin-supplemented agar plates. PA14 and JE2 were cultured on nutrient gelatin agar and incubated at 37 °C, alongside nonproteolytic control strains (*Staphylococcus epidermidis* 1457 and *Escherichia coli* K12). Following incubation, plates were treated with saturated ammonium sulfate to precipitate undegraded gelatin, and clear zones of gelatin hydrolysis were used as an indicator of protease activity. Representative images were acquired using a ChemiDoc MP imaging system.

### 2.4 CFU Quantification

To assess bacterial retention on film surfaces, colony-forming unit (CFU) enumeration was performed following incubation with *P. aeruginosa* PA14. Film samples were incubated with bacterial suspensions for 12 h under static conditions. After incubation, films were gently rinsed with sterile PBS to remove nonadherent cells, transferred to fresh PBS, and subjected to sonication for 5 min followed by vortexing for 30 s to detach surface-associated bacteria. The resulting suspensions were serially diluted in PBS and plated on LB agar. Plates were incubated at 37 °C for 18–24 h, after which colonies were counted and CFU values were calculated.

### 2.5 Fourier Transform Infrared-Attenuated Total Reflectance Spectroscopy (FTIR-ATR)

Chemical composition and molecular interactions within films were analyzed using attenuated total reflectance FTIR spectroscopy (Bruker Tensor 27) over a range of 4000–500 cm^–1^ with 4 cm^–1^ resolution and 32 scans. Film samples (approximately 1 cm × 1 cm) were placed directly on the ATR crystal. Background spectra were collected before each measurement and automatically subtracted. All measurements were performed at room temperature and spectra were baseline-corrected using OPUS software (Bruker).

Peak intensity reductions in the amide *I* region were calculated according to eq 1

1 $$i\mathrm{ntensity reduction}\;\left(\%\right)=\frac{\left(I_{\mathrm{PA}14,\mathrm{amide I}}-I_{\mathrm{PA}14,\mathrm{baseline}}\right)-\left(I_{\mathrm{LB},\mathrm{amide}I}-I_{\mathrm{LB},\mathrm{baseline}}\right)}{\left(I_{\mathrm{LB},\mathrm{amide}I}-I_{\mathrm{LB},\mathrm{baseline}}\right)}\times 100$$

where *I*_PA14,amideI_ and *I*_LB,amideI_ are the amide *I* peak intensities of bacterial-exposed and LB-treated control samples, respectively. Baseline absorbance was defined at 1787.7 cm^–1^, corresponding to the first wavenumber beyond the amide *I* region where the spectrum exhibited a consistently positive first derivative up to the amide *I* maximum. This formulation normalizes intensity changes to corresponding controls and accounts for baseline offsets and thickness variations.

Prior to measurements, bacterial-exposed films were decontaminated by sequential rinsing in PBS, 70% ethanol, and PBS, then fully dried.

### 2.6 Swelling and Dissolution Measurements

Films were weighed to obtain initial dry weights (*W*_initial), then immersed in sterile nutrient media or bacterial cultures in 12-well plates at 37 °C for specified durations in static conditions. For swelling measurements, films were removed, excess surface liquid was gently blotted with filter paper, and swollen weights (*W*_swollen) were immediately recorded. Films were then dried at 37 °C for 24 h and final dry weights (*W*_dry) were measured. Swelling degree was calculated as

$$s\mathrm{welling degree}\;\left(\%\right)=\frac{W_{\mathrm{swollen}}-W_{\mathrm{dry}}}{W_{\mathrm{dry}}}\times 100\%$$

and dissolution degree as

$$d\mathrm{egree of}\;d\mathrm{issolution}\;\left(\%\right)=\frac{W_{\mathrm{initial}}-W_{\mathrm{dry}}}{W_{\mathrm{initial}}}\times 100\%$$

Three independent biological experiments were performed on separate days, with three technical replicates for each experimental condition.

### 2.7 Scanning Electron Microscopy

Surface morphology was examined using a TM3030Plus tabletop scanning electron microscope (Hitachi, Japan). Following bacterial exposure, samples were gently rinsed sequentially with deionized water, ethanol, and deionized water to remove residual culture medium and nonadherent bacteria prior to imaging. Samples were mounted on aluminum stubs using double-sided conductive carbon tape and imaged at 15 kV accelerating voltage.

Cryogenic SEM imaging was performed to visualize bacteria-material interactions and degradation in the hydrated state following bacterial exposure, therefore samples were imaged after 12 h exposure to ensure adequate colonization and hydrogel formation. Film samples exposed to bacteria were fixed with 4% paraformaldehyde for 15 min, rinsed with PBS, and stored at 4 °C until processing. Samples were mounted on specimen stubs and rapidly frozen using nitrogen slush plunge-freezing. Following fracturing, samples were sublimated at −90 °C for 5–10 min and sputter-coated with platinum. Images were captured using a JEOL JSM-IT800 SEM equipped with a cryogenic system at 3.0 kV accelerating voltage while maintaining frozen conditions. Pore size analysis was performed using ImageJ software on multiple fields of view.

### 2.8 Quartz Crystal Microbalance with Impedance (QCM-I) Measurements

To capture degradation kinetics with nanogram sensitivity, QCM-I (QCM-I Micro system, Semilab Co. Ltd., Budapest, Hungary) was used to monitor frequency and bandwidth changes. Compared to end point assays, QCM-I resolves the onset and pace of proteolysis in real time while distinguishing mass loss from viscoelastic softening at the interface. Prior to each measurement, gold-coated quartz sensors were cleaned by UV/ozone treatment for 10 min, followed by immersion in heated cleaning solution (75 °C) containing water, ammonia (25%), and hydrogen peroxide (30%) in a 5:1:1 ratio for 5 min, then rinsed, UV/ozone treated for 5–10 min, and dried.^50^

Films were deposited onto cleaned sensors by spin-coating. For GG coatings, 150 μL of gelatin-GPTMS solution was dispensed and spun at 500 rpm for 2 s, 2000 rpm for 1.5 s, and 5000 rpm for 30 s. For GG:PEO coatings, solutions were diluted 2-fold and spun at 1000 rpm for 5 s, 2000 rpm for 1.5 s, and 5000 rpm for 2 min to accommodate higher viscosity. Coated sensors were cured at 80 °C for 30 min.

For enzymatic degradation studies, *P. aeruginosa* PA14 cultures at OD_600_ = 0.05 were incubated statically at 37 °C for at least 12 h. Culture supernatants were collected, centrifuged at 4000 rpm for 5 min, and filter-sterilized through 0.22 μm filters to obtain cell-free enzymatic solutions. QCM measurements were conducted at 37 °C using a benchtop incubator to maintain physiological temperatures. Baseline measurements were established in air and sterile LB medium, then enzymatic solutions were introduced via syringe pump while continuously monitoring frequency and bandwidth changes at the fundamental resonance and overtones (3rd, 5th, 7th, 9th). Initial degradation rates for GG100 and GG75:PEO25 coatings were calculated as the linear slope immediately after enzyme introduction (3rd overtone, Hz/min).

For quantitative analysis of rigid, thin, and uniform films that are tightly coupled to the sensor (Sauerbrey limit), Sauerbrey equation was measured as eq 2

2 $$\Delta f=-\frac{2f_{0}^{2}}{A\sqrt{\rho_{q}E_{q}}}\Delta m=>\Delta f\propto \Delta m$$

where *A* is the active area and ρ_q_ and *E*_q_ are the density and Young’s modulus of quartz. For viscoelastic films, the calculated Sauerbrey mass was considered to be reasonable representation of the rigidity coupled mass of a film as long as the accompanying change in bandwidth is less than minus half the frequency change (0 < |ΔFWHM/Δ*f*| < 0.5). By comparison, for a pure viscosity change above the sensor surface ΔFWHM/Δ*f* = −2.

Mammalian cell-conditioned media were obtained from A549 human epithelial cells cultured under standard conditions. Cell density was determined prior to collection, and the supernatant corresponded to a concentration of approximately 1 × 10^6^ cells mL^–1^. To remove intact cells and cellular debris, the collected culture medium was passed through a sterile syringe filter prior to QCM measurements. The resulting filtered supernatant was used directly without further dilution. For QCM experiments, a blank cell culture medium was first circulated to establish a hydrated baseline before introduction of the filtered A549-conditioned medium.

To assess the contribution of active proteolytic enzymes to the observed QCM responses, bacterial supernatants were treated with protease inhibitors prior to measurement. PA14 supernatants were supplemented with a commercially available protease/phosphatase inhibitor cocktail (100×, Thermo Fisher Scientific) and EDTA to inhibit both serine proteases and metalloproteases. Specifically, PA14 supernatant was combined with 1× inhibitor cocktail and 10 mM EDTA. The inhibited supernatants were incubated at room temperature for 15–20 min immediately prior to QCM measurements and then introduced into the QCM flow cell without further modification. A blank LB medium control was measured prior to inhibited supernatant exposure to establish the hydrated baseline.

### 2.9 Statistical Analysis

Data are presented as mean ± standard deviation from at least three independent biological replicates unless otherwise specified. Statistical comparisons between two groups were performed using unpaired Student’s *t* tests after confirming normal distribution. Multiple group comparisons were analyzed by two-way ANOVA followed by appropriate posthoc tests. Linear regression was performed to assess correlations between variables. Data were significant when *p* < 0.05. All statistical analyses were performed using GraphPad Prism (version 10.6).

## 3 Results and Discussion

### 3.1 Cross-Link Density Modulates Enzymatic Degradation at the Molecular Level

The design of enzyme-responsive materials for bacterial sensing requires careful balance between structural stability and enzymatic accessibility. Prior to these characterizations, extracellular protease activity of PA14 and JE2 under the experimental conditions was verified using gelatin-supplemented agar plates. Both strains produced clear zones of gelatin degradation, while nonproteolytic control strains showed no detectable clearance (Figure S1).

To establish the optimal cross-linking density for our gelatin-based sensing platform, we employed FTIR-ATR spectroscopy to investigate how GPTMS concentration influences the molecular response of gelatin films to bacterial proteolytic activity. The gelatin-GPTMS (GG) films investigated here were extensively characterized in our previous work, where the influence of GPTMS cross-linker density on network formation and chemical structure was systematically quantified.^51^ In the present work, we leverage this established material framework and focus on how cross-linker density influences the behavior of GG films under bacterial enzymatic activity.

Three gelatin formulations with varying cross-link densities were prepared using gelatin-to-GPTMS mass ratios of 1:10, 2:10, and 3:10, denoted as GG1:10, GG2:10, and GG3:10 respectively. These represent progressively denser cross-linked networks where increasing GPTMS content creates more siloxane bridges between gelatin chains. FTIR spectra of control films maintained in sterile LB medium showed remarkable stability across all time points and formulations (Figure 2). The characteristic amide bands, amide A (N–H stretching, ∼3290 cm^–1^), amide I (C=O stretching, ∼1630 cm^–1^), amide II (N–H bending coupled with C–N stretching, ∼1540 cm^–1^), and amide III (C–N stretching and N–H bending, ∼1240 cm^–1^), were identified with consistent intensities throughout the monitoring period. This consistency confirmed that the aqueous medium alone did not induce chemical degradation of the gelatin matrix, and that any subsequent changes observed in bacterial-exposed samples could be attributed specifically to enzymatic activity.

**Figure 2:**
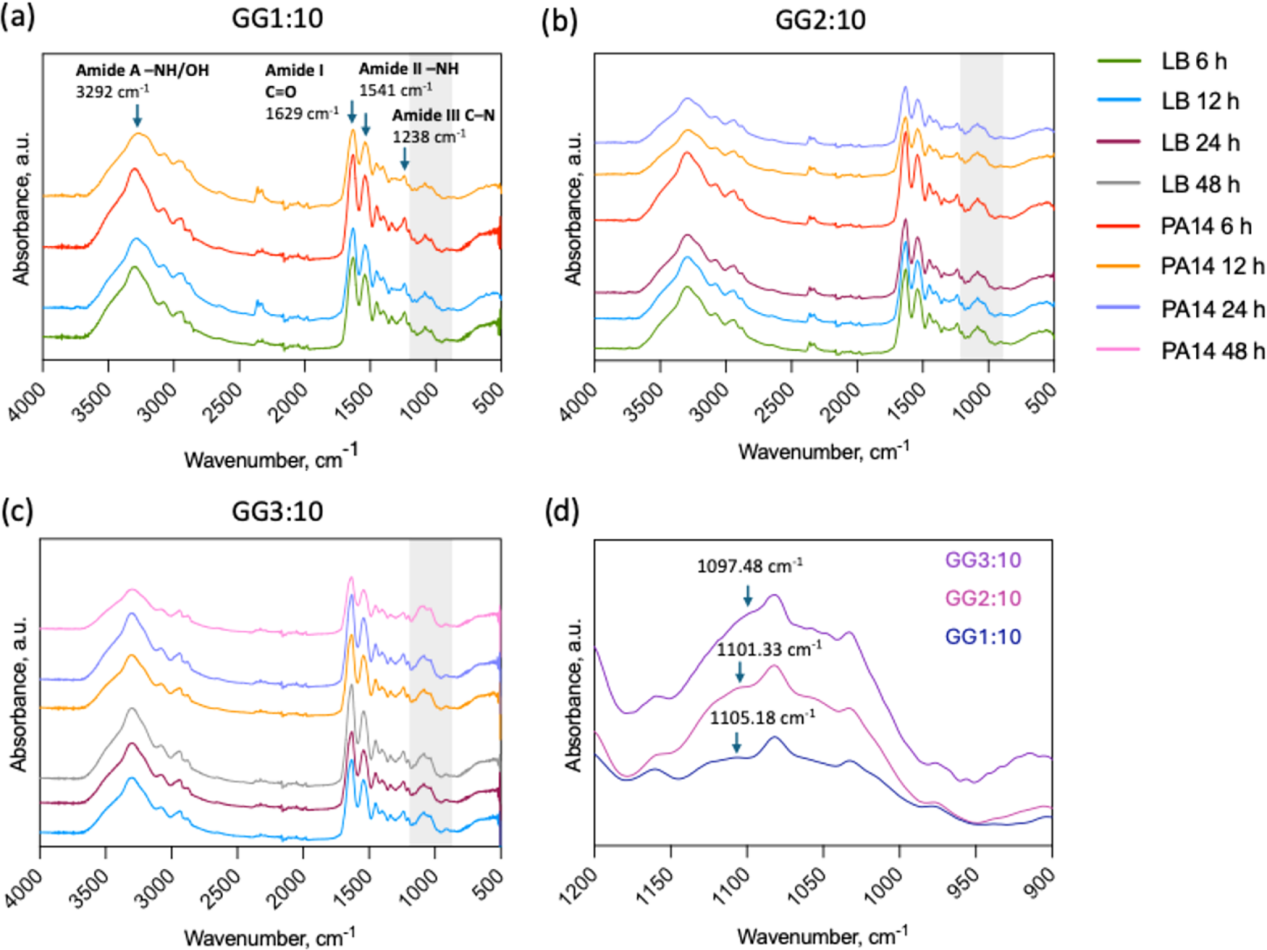
FTIR-ATR spectra for (a) GG1:10, (b) GG2:10, and (c) GG3:10 films exposed to control LB medium and *P. aeruginosa* PA14 for 6–48 h. Each color represents a sample exposed to either LB medium or PA14 at different time points. Arrows indicate the characteristic peaks for amide bands. Shaded region shows the area attributed to siloxane cross-link bonds. (d) Spectra at 1200–900 cm^–1^ for GG1:10, GG2:10, GG3:10 films exposed to *P. aeruginosa* PA14 for 12 h. Arrows indicate the shoulder at around 1100 cm^–1^ corresponding to Si–O–Si bonds.

Upon exposure to a common wound pathogen *P. aeruginosa* (PA14) cultures, the FTIR spectra underwent progressive and composition-dependent changes. The most diagnostic alterations occurred in the amide I and amide II regions, where the intensity decreased substantially over time in all formulations. In addition, Amide A region (∼3300 cm^–1^) demonstrated broadened peaks after bacteria exposure which could indicate increased hydrogen bonding heterogeneity and peptide disorder. For GG1:10 films, amide I intensity showed measurable reduction as early as 6 h, with dramatic decreases by 12 h (Table S1). GG2:10 exhibited similar but temporally delayed reductions with complete gelatin loss after 48h, while GG3:10 maintained significant amide I intensity even after 48 h of exposure. These intensity decreases directly suggested the loss of peptide bonds through hydrolytic cleavage catalyzed by bacterial proteases, particularly the gelatinases known to be secreted by PA14.

Importantly, while amide bands attenuated and slightly broadened with bacterial exposure, the Si–O–Si stretching vibration at approximately 1100 cm^–1^ (highlighted area in Figure 2a–c) remained unchanged across all samples. This observation suggested that the GPTMS-derived siloxane network remains chemically intact during enzymatic degradation.

Although the siloxane bond intensity remained unchanged compared to blank samples, as the cross-link density increased from GG1:10 to GG3:10, the Si–O–Si stretching frequency progressively shifted to lower wavenumbers from ∼1105 cm^–1^ to ∼1097 cm^–1^ after PA14 exposure for 12 h (shown in Figure 2d). This behavior can be interpreted by the siloxane network geometry. According to the central force model for Si–O–Si vibrations, the stretching frequency depends on the intertetrahedral bond angle, which in turn reflects the local structural environment around each siloxane linkage.^52^ As gelatin fragments were enzymatically removed at lower cross-link densities, the remaining siloxane network likely underwent structural relaxation and densification, adopting more thermodynamically favorable bond angles characteristic of condensed silica (observed higher wavenumbers for GG1:10). This is consistent with network relaxation and change in intertetrahedral geometry within the siloxane domains during peptide removal. We therefore interpret the observed frequency shifts as one possible indicator of changes in the local siloxane environment associated with enzymatic exposure, although this interpretation cannot be confirmed using this model alone.

The spectral changes reflected chain scission rather than complete material removal as the cross-linker peak intensities remained unchanged in the PA14 exposed films. This suggested that the cleaved peptide fragments initially remained within the film matrix before potential leaching occurred that resulted in the complete dissolution of the gelatin network. Based on the observed behavior at the molecular level, a physical model was proposed through a two-stage process: (1) enzymatic cleavage of peptide bonds created smaller, more mobile gelatin fragments, followed by (2) leaching of these cleaved fragments from the cross-linked network into the surrounding medium. The siloxane network therefore remains chemically intact but loses mechanical integrity as the gelatin component is progressively removed.

Importantly, the cross-link density determined the extent of fragment retention. According to the proposed model, GG1:10 films with sparse cross-links allow extensive fragment removal leading to network collapse and complete dissolution, GG2:10 films with intermediate cross-linking retain sufficient fragments to maintain structural integrity despite partial degradation at later time points, and GG3:10 films with dense cross-linking act as a molecular cage that prevent fragment leaching despite peptide bond cleavage, explaining the minimal spectral features even after extended enzymatic exposure.

To further assess the influence of GPTMS cross-linking on network structure, swelling behavior of GG films with varying cross-linker content was quantified following exposure to PA14 enzymatic environment (Figure S2). Increasing GPTMS content resulted in a systematic reduction in swelling ratio, consistent with increased network density and reduced chain mobility. Exposure to bacterial enzymes partially restored swelling in lower-cross-linked networks (GG1:10), whereas highly cross-linked films showed significantly constrained swelling, indicating enhanced resistance to enzymatic disruption. These trends are consistent with FTIR evidence of progressive siloxane network formation and support the role of GPTMS cross-linking in governing enzymatic accessibility and degradation kinetics.

Based on these molecular-level observations, the intermediate GG2:10 formulation offered the most promising balance of properties by retaining sufficient structural integrity for practical handling while demonstrating progressive degradation in response to bacterial proteolytic activity over 48 h. This formulation is referred to as GG100 in subsequent sections to highlight its gelatin content. However, the 48 h time frame required for substantial degradation presents a critical limitation for clinical utility, where rapid infection detection within 12–24 h is essential for timely intervention and improved patient outcomes. To address this challenge, we designed hybrid films incorporating poly­(ethylene oxide) as a hydrophilic polymer component specifically selected to accelerate degradation kinetics, while maintaining the selective degradation mechanism validated through the FTIR analysis.

### 3.2 Influence of PEO Content on Film Hydration and Proteolytic Activity Response

To achieve clinically relevant detection timeframes, we developed gelatin-PEO hybrid films where PEO serves as an additional modulator for enzymatic degradation. By systematically varying PEO content from 15% to 40% in the solvent-cast films, we designed a series of formulations and investigated the tunability of their degradation rates over time, potentially addressing the sensitivity-stability balance for different clinical scenarios.

Swelling studies of GG:PEO hybrid films immediately showed that PEO content strongly affects hydration behavior (Figure 3). While the GG2:10 base formulation swelled to <150% in sterile LB medium, incorporation of just 15% PEO in the solvent-cast films increased equilibrium swelling to approximately 350%, and 40% PEO drove swelling ratios to nearly 800%, suggesting expanded water-rich mesh that helps enzyme diffusion. These dramatic increases reflected the ability of PEO to form extensive hydrogen-bonding networks with water molecules. The hydration enhancement fundamentally altered the physical state of the films, transforming them from relatively compact hydrogels into highly swollen structures with significantly increased mesh size and permeability.

**Figure 3:**
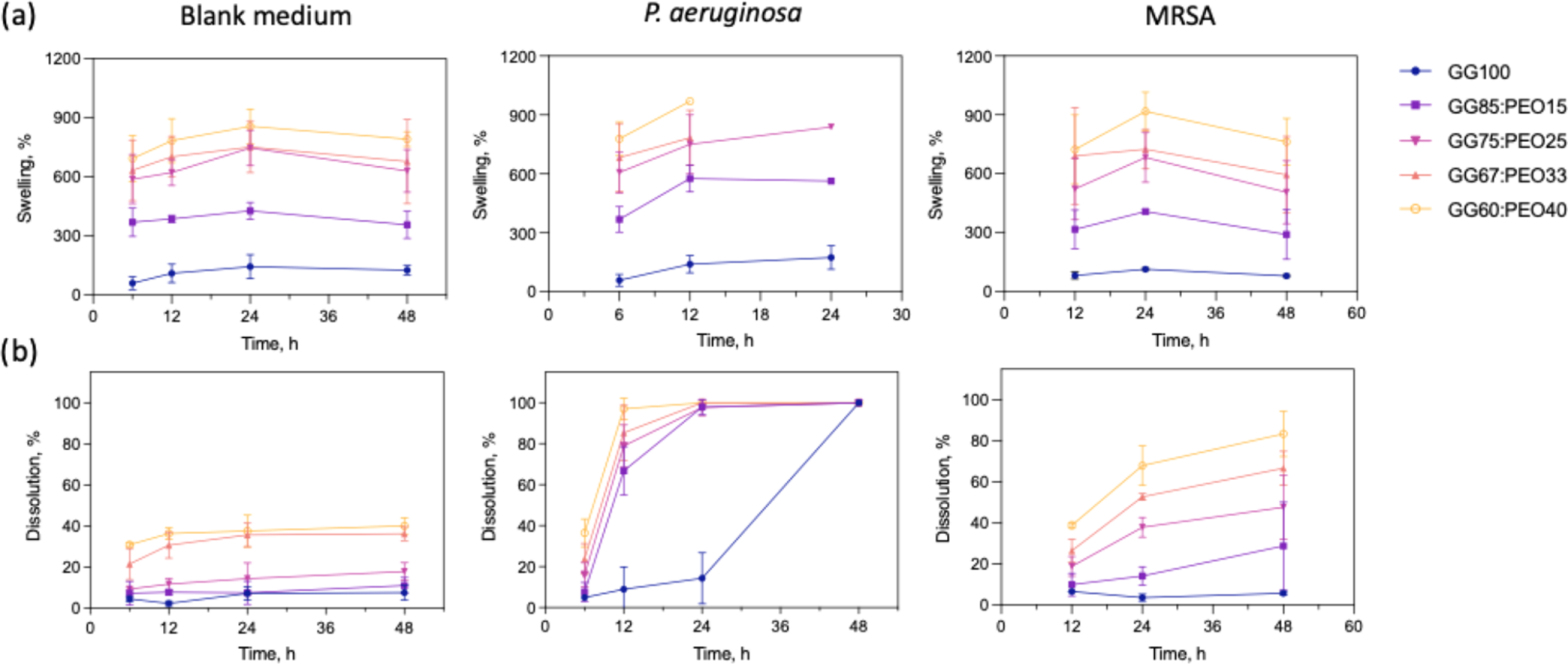
Time-dependent (a) swelling and (b) dissolution percentages of GG:PEO films with varying compositions when exposed to blank medium (control), *P. aeruginosa*, and MRSA cultures. Swelling rates were calculated up to the time point where films were physically handleable. Data presented as average ± standard deviation (*N* = 3 biological replicates). Statistical significance for swelling data was determined by two-way ANOVA among the compositions and time for blank medium (*p* < 0.0001 composition, *p* = 0.0077 time), and MRSA (*p* < 0.0001 composition, *p* = 0.0715 time). It was not possible to fit a full ANOVA model to *P. aeruginosa* data, as some data points were missing due to dissolution.

The impact of PEO on enzymatic susceptibility became highly apparent in dissolution measurements (Figure 3b). Under sterile conditions, all GG:PEO formulations showed some degree of dissolution that increased with PEO content, indicating that PEO incorporation slightly reduced the intrinsic stability of the cross-linked network even in the absence of enzymatic activity. This baseline dissolution likely resulted from gradual leaching of loosely bound PEO chains and gelatin fragments that were insufficiently immobilized by the cross-linking process.

Upon exposure to PA14 cultures, the dissolution kinetics accelerated dramatically. Films containing 33–40% PEO underwent nearly complete degradation within 12 h, while even the GG85:PEO15 formulation lost more than 70% of its mass by 24 h. In contrast, the pure GG100 composition retained most of its structure through this time frame. Analysis of the initial degradation rate slopes from 6–12 h showed approximately 15-fold acceleration for GG60:PEO40 compared to GG100, confirming that PEO incorporation dramatically enhanced enzymatic susceptibility.

Visual assessment of the films over time provided qualitative confirmation of these accelerated kinetics (Figure S3). GG60:PEO40 films showed visible structural deterioration within 6 h of PA14 exposure, with obvious thinning and loss of optical clarity indicating substantial material loss. By 12 h, these high-PEO films had largely disintegrated, leaving only fragmented remnants. Lower PEO concentrations displayed more controlled degradation, with eventual dissolution occurring over 12–24 h. The GG75:PEO25 formulation emerged as particularly interesting in this regard, showing measurable degradation at early time points while maintaining sufficient structural integrity to be handled and characterized throughout the initial 12-h period.

Having established the GG:PEO degradation profiles when exposed to *P. aeruginosa*, we next examined whether this platform would demonstrate similar utility for detecting other clinically important wound pathogens with different enzymatic profiles. Methicillin-resistant *S. aureus* (MRSA) represents another critical target, as it accounts for substantial fractions of chronic wound infections and produces a distinctly different proteolytic enzyme arsenal compared to *P. aeruginosa*.^53,54^

Dissolution studies with MRSA strain JE2 revealed degradation kinetics substantially slower than those observed with PA14, though still clearly distinguishable from sterile controls (Figure 3 right column). The GG60:PEO40 formulation, which had disintegrated within 12 h when exposed to PA14, required 24 h to reach approximately 70% dissolution with MRSA and only achieved approximately 85% mass loss by 48 h. The GG75:PEO25 composition showed even more dramatic temporal differences, losing only about 30% by 24 h and approximately 55% by 48 h with MRSA, compared to complete dissolution at these same time points with PA14. These slower kinetics can be rationalized by differences in the proteolytic systems of the two species. *P. aeruginosa* secretes high levels of elastase (LasB), a zinc metalloprotease with broad substrate range and strong proteolytic activity,^55^ whereas *S. aureus* secretes proteases such as aureolysin and the Spl family, which exhibit narrower substrate specificities and lower overall activity toward gelatin.^56^ Moreover, kinetic differences suggest that the timing and magnitude of enzyme secretion may differ between the species, with *P. aeruginosa* releasing large quantities of extracellular proteases early during active growth, and *S. aureus* producing them more gradually.

Importantly, despite these kinetic differences, MRSA-induced degradation showed clear PEO-dependence, with higher PEO compositions degrading more rapidly than lower-PEO formulations. This confirmed that the same fundamental mechanisms operate regardless of specific bacterial species or enzyme mixture present. This pathogen-independent mechanism represented a significant advantage for potential clinical use, as wound infections are frequently if not universally polymicrobial. A sensing platform responding to total proteolytic activity from diverse pathogens through a common mechanism offers broader utility than approaches targeting species-specific biomarkers.

The acceleration in degradation rate due to PEO incorporation can be rationalized through several mechanisms operating at different scales. At the molecular level, PEO chains likely increased the free volume within the polymer network and disrupted the packing of gelatin chains, creating a more open structure with enhanced permeability to macromolecules including bacterial proteases. At the nanoscale, the extensive hydration facilitated by PEO reduced the effective concentration of gelatin chains and increased the mesh size of the network, allowing enzymes to diffuse more rapidly through the matrix and access more cleavage sites per unit time. At the microscale, PEO-rich domains may create pathways for both enzyme accessibility and fragment removal, accelerating mass transport processes. Nevertheless, the fact that adding PEO reduces the total amount of cross-linker in the film should also be considered. This is due to two factors: (1) lower percentage of cross-linker, and (2) PEO is a part of the film that does not contribute to the cross-linked network.

The trade-off between enhanced responsiveness and structural stability became increasingly apparent as PEO content increased when exposed to both pathogens. While the accelerated degradation of high-PEO formulations (33–40%) might appear advantageous for rapid detection applications, the accompanying loss of mechanical integrity and the substantial baseline dissolution in sterile medium compromised their practical utility. Films must maintain sufficient structural stability during storage, handling, and deployment to wound sites, and must provide clear discrimination between bacterial and sterile conditions. Formulations with excessive PEO content failed these criteria particularly under rapid *P. aeruginosa* activity, therefore providing limited temporal resolution of degradation kinetics. In contrast, GG75:PEO25 was selected for further characterization as a formulation that provided reliable function across both rapid and slow degradation scenarios, offering versatility for detecting diverse wound pathogens.

### 3.3 Morphological Characterization of Enzyme-Exposed Films

While molecular spectroscopy established the chemical basis for degradation and gravimetric measurements quantified mass loss, neither technique directly visualizes the structural transformations occurring within the degrading network. To bridge the gap between molecular changes and macroscopic properties, we employed scanning electron microscopy to characterize the GG75:PEO25 films, our selected formulation, at progressive degradation stages.

As shown in Figure 4, conventional SEM analysis of GG75:PEO25 films showed significant and strain-dependent morphological changes compared to sterile controls and the unexposed 0 h controls shown in Figure S4. Control films maintained in sterile LB medium displayed relatively uniform surface topography with some phase-separated features characteristic of the hybrid gelatin-PEO network.^57,58^

**Figure 4:**
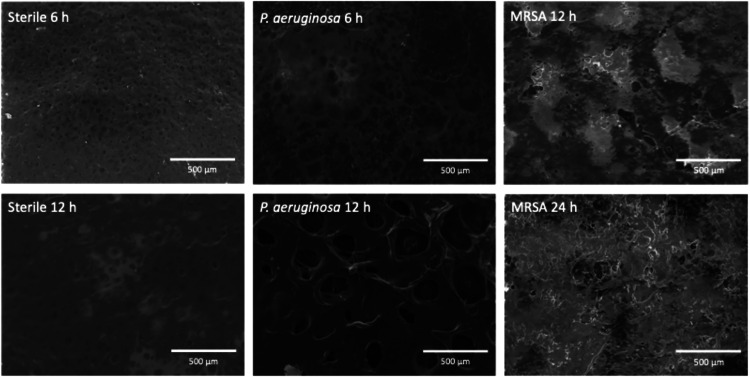
Representative SEM micrographs of GG75:PEO25 films exposed to sterile LB media (left column), *P. aeruginosa* culture (middle column) for 6 and 12 h, and MRSA culture (right column) for 12hand 24 h due to the slower degradation kinetics. Scale bars indicate 500 μm.

Following *P. aeruginosa* exposure for 6 h, phase separation visibly increased, indicating the compromised gelatin network structure due to the enzymatic activity as gelatin fragments were removed and PEO domains became more apparent and densely aggregated. At 12 h, the surface morphology transformed into a network of large pores, suggesting more extensive structural disruption. The average pore diameter reached 74.4 ± 35.7 μm, with the remaining polymer appearing as thin stretched membranes between voids that looked close to mechanical failure. This porous architecture after 12 h directly suggested the selective enzymatic removal of gelatin chains, consistent with the approximately 80% mass loss measured at this time point. The heterogeneous pore size distribution indicated nonuniform degradation, with some regions undergoing extensive material removal while others retained partial structure, likely due to local variations in cross-link density or enzyme accessibility.

In contrast, films exposed to MRSA exhibited a more gradual morphological evolution, and SEM images are therefore shown at later time points (12 and 24 h) prior to complete film dissolution. At 12 h, MRSA exposure resulted in heterogeneous surface erosion and localized roughening without the extensive pore formation observed for PA14. By 24 h, widespread surface degradation and loss of structural integrity were evident, which was also consistent with the slower degradation behavior of MRSA inferred from bulk dissolution measurements.

However, conventional SEM requires sample drying that can induce artifacts including pore collapse and polymer reorganization. To reliably visualize the hydrated-state morphology, we performed cryogenic SEM on frozen-hydrated GG75:PEO25 samples, which preserves structures much closer to their native aqueous conformations. Control samples maintained in sterile LB for 12 h revealed the intrinsic architecture of the swollen network as a highly porous foam-like structure with small and relatively uniform pores averaging 0.39 ± 0.22 μm in diameter (Figure 5a). This submicron baseline porosity represented the mesh structure of the hydrated gel through which enzymes must diffuse and fragments must escape.

**Figure 5:**
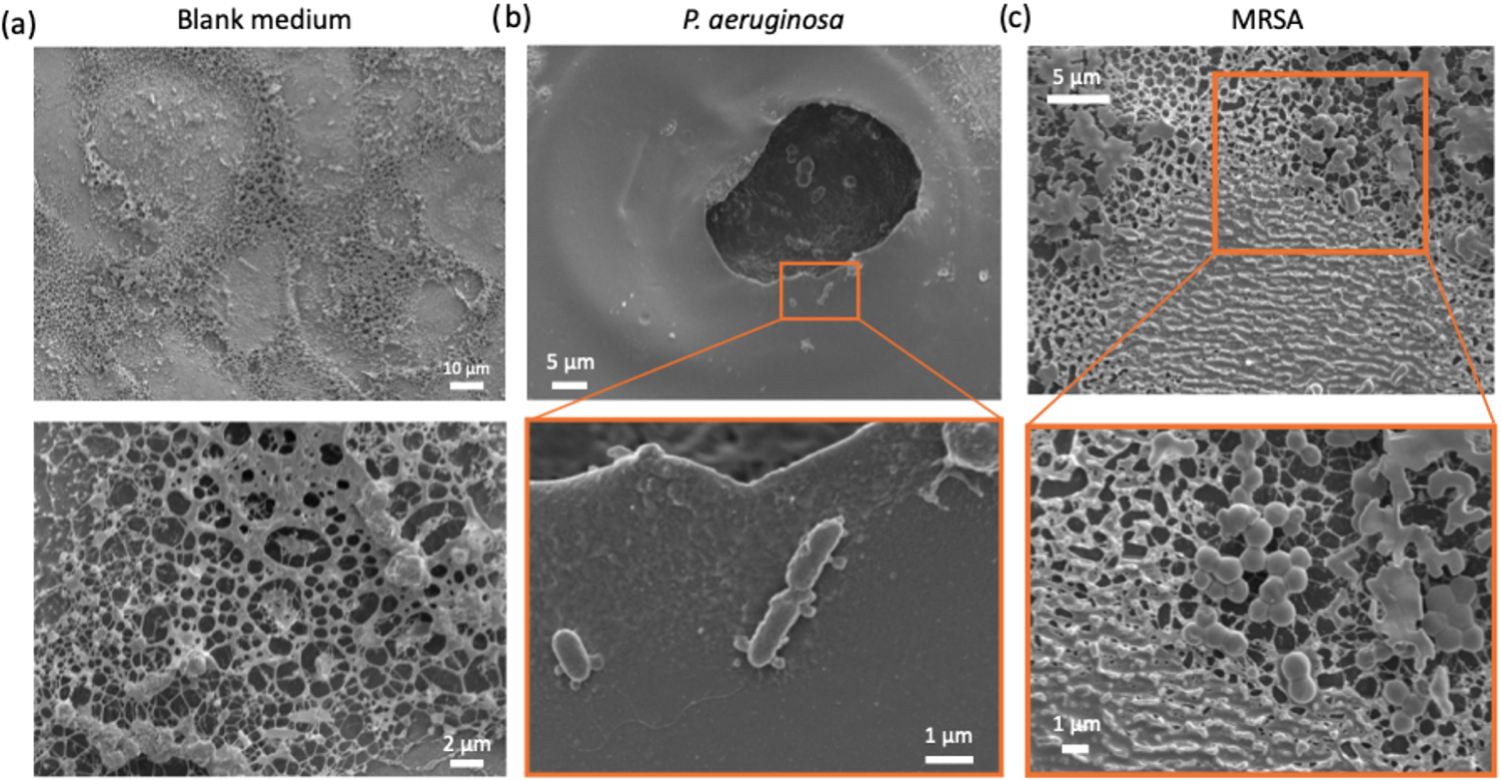
Representative cryogenic SEM micrographs of GG75:PEO25 films exposed to (a) sterile LB medium, (b) *P. aeruginosa*, and (c) MRSA cultures for 12 h. Images on the bottom show selected areas of the top images with higher magnifications.

Following 12 h of PA14 exposure, cryo-SEM showed individual rod-shaped bacterial cells (approximately 1–2 μm length) adhering sparsely to the film surface, rather than forming the dense biofilms typically expected from *P. aeruginosa* (Figure 5b). The surrounding polymer matrix displayed highly irregular surfaces with pit-like formations and obvious material loss, indicating material degradation.

The limited bacterial adhesion, despite clear enzymatic activity, was likely due to two factors. First, the surface may have antiadhesive properties. PEO is well-documented for showing protein and biomolecule adhesion resistance due to its steric repulsion effects.^200,201^ The high swelling ratios reported in the previous section (400–600%) could be the indicator of a highly hydrated surface layer that prevents stable bacterial attachment. To examine the first hypothesis, bacterial retention on GG and GG75:PEO25 films was quantified using colony counting following rinsing and detachment (Figure S5). Under the conditions tested, no statistically significant difference in CFU counts was observed between GG and GG:PEO films, suggesting that the presence of PEO did not measurably reduce overall bacterial attachment on the films. Therefore, a more plausible hypothesis is that the enzymatic degradation may paradoxically limit bacterial colonization. As *P. aeruginosa* secretes proteases that degrade the gelatin matrix, it may be destroying its own adhesion substrate, therefore, preventing the establishment of stable microcolonies necessary for biofilm development. As the data reported in Figure 3 also suggested substantial mass loss (66–96% at 12 h for GG75:PEO25), we therefore believe that progressive dissolution promotes bacterial detachment from the surface as the substrate dissolves beneath them.

By contrast, surprisingly, MRSA JE2 formed dense clusters (approximately 0.8–1.0 μm diameter) with biofilm matrix after 12 h (Figure 5c), consistent with slower, more controlled proteolysis that preserves the scaffold long enough for colonization. As discussed in previous sections, MRSA likely demonstrated slower and more controlled proteolytic activity, which preserved the film structure long enough to support biofilm establishment. The intact matrix likely served as a scaffold for biofilm expansion, whereas *P. aeruginosa* aggressive enzymatic degradation destroyed its own substrate before stable biofilm formation could occur. Additionally, MRSA expresses multiple surface adhesins (e.g., fibronectin-binding protein A/B) capable of binding gelatin, as well as polysaccharide intercellular adhesin (PIA), both contributing to strong and persistent attachment.^59,60^ These mechanisms could resist the potential antiadhesive properties of the PEO surface.

SEM observations provided several critical insights. First, they confirmed that hydrated GG75:PEO25 films undergo dramatic structural transformation during enzymatic activity, with pore sizes increasing from submicron baseline values to tens of microns, representing roughly 100-fold expansion that eventually leads to network collapse. Second, the direct visualization of viable, metabolically active bacteria on film surfaces established that observed degradation reflects genuine bacteria-material interactions. Third, the pathogen-specific colonization behaviors, as sparse *P. aeruginosa* adhesion versus denser MRSA biofilms, indicated complex interactions where degradation kinetics and bacterial behavior influence each other, offering potential for not just bacterial detection but partial pathogen discrimination based on combined degradation rates and colonization patterns.

### 3.4 Real-Time Monitoring of Enzymatic Degradation Using Quartz Crystal Microbalance

Having established the composition-property relationships governing enzymatic degradation through end point measurements, we sought to probe the degradation process with higher temporal resolution and nanogram-scale sensitivity using quartz crystal microbalance with impedance analysis (QCM-I). While the previous characterization techniques provided valuable snapshots of degradation at discrete time points, they could not capture the dynamic evolution of film properties during enzymatic activity. QCM-I addresses this limitation by enabling continuous, real-time monitoring of both mass changes and viscoelastic transitions at the film-sensor interface, providing information on how enzymatic cleavage progressively disrupts network structure and mechanical properties.

Before examining enzymatic degradation, we first characterized the baseline behavior of our coatings under air and aqueous conditions to establish reference states and validate the measurement approach. Films of cross-linked gelatin (GG100) and GG75:PEO25 were spin-coated onto gold-coated quartz sensors, and their frequency and bandwidth responses were monitored during transitions from air to liquid environments at room temperature. These baseline measurements provided essential context for interpreting subsequent bacterial exposure experiments and are presented in Figure S6. Several key observations emerged from these studies. Both GG100 and GG75:PEO25 coatings showed stable signals in air with minimal bandwidth changes, confirming rigid, elastic films. The dry film thicknesses calculated from the Sauerbrey equation^61^ using estimated density values were approximately 217 nm for GG100 and 213 nm for GG75:PEO25. Upon immersion in aqueous buffer, both coatings exhibited characteristic hydration responses, as frequency decreases and bandwidth increases, that indicated network swelling and the transition from rigid to viscoelastic behavior. Importantly, GG100 coatings maintained relatively rigid character even when hydrated, with the ∼116% increase in mass or thickness. This is supported by the Δ*f*_1_ and Δ*f*_3_/3 changes being very similar to each other. In contrast, the GG75:PEO25 film displayed more pronounced viscoelastic behavior with larger bandwidth increases indicative of greater chain mobility and water incorporation. The apparent “Sauerbrey mass” increase for GG75:PEO25 was much lower than for GG100, despite greater swelling measured above. The large bandwidth increase and trajectory position near the viscous limit in ΔFWHM vs Δ*f* plot indicated that the hydrated GG:PEO film becomes highly viscous, with part of its mass decoupled from the sensor surface. This decoupling was further confirmed by the divergent behavior between the fundamental and third overtone frequencies. In this context, the Sauerbrey mass can be seen to represent the amount of the film mass that is still acoustically coupled to the sensor surface, and a reduction could therefore be due to loss of material from the surface, or loss of rigidity of the film.

With these reference states established, we examined how exposure to bacterial enzymatic solutions affected the coatings in real time. To isolate enzymatic effects from bacterial adhesion, we prepared cell-free supernatants from PA14 biofilms through centrifugation and sterile filtration. These supernatants contained the secreted proteolytic enzymes without the complicating factors of ongoing bacterial growth or cellular adhesion. Measurements were performed at 37 °C to match physiological temperatures relevant for wound environments. After coating the dry films, the experimental protocol consisted of establishing a stable baseline with the coated sensor in sterile LB medium, allowing the film to reach equilibrium hydration, then exchanging the medium for PA14 enzymatic supernatant while continuously monitoring frequency and bandwidth changes across multiple overtones. At 37 °C, both films showed significantly increased viscoelastic behavior due to the increased flexibility of the gelatin backbone within the cross-linked films at the elevated temperature, which is above the melting point of the native gelatin at the used concentrations.

Real-time frequency and bandwidth monitoring revealed the dynamic response of both coatings to enzymatic exposure (Figure 6). Both GG100 and GG75:PEO25 showed characteristic frequency decreases and bandwidth increases upon hydration, followed by frequency increases and bandwidth decreases upon enzymatic exposure. Based on the Sauerbrey interpretation discussed above, immediately following hydration, increasing frequency indicated progressive decoupling of the swollen gel structure from the sensor rather than simple mass removal, as the softened network becomes less rigidly attached to the crystal.

**Figure 6:**
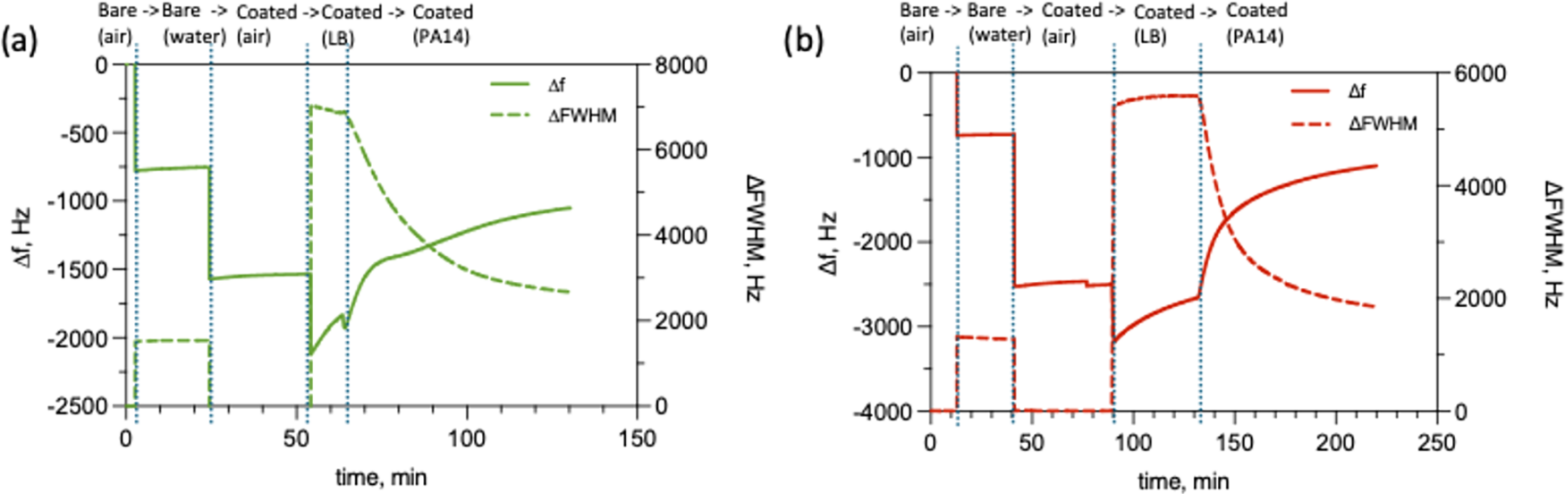
Fundamental resonance frequency and dissipation changes through ΔFWHM for (a) GG100 and (b) GG75:PEO25 coatings after PA14 enzymatic exposure. Left axes show Δ*f* and right axes present ΔFWHM values. Each experimental step over time is indicated at the top of the plots and is separated using blue dashed lines.

Upon introduction of PA14 enzymatic solution, both coatings showed more dramatic responses. Frequency began increasing while bandwidth simultaneously decreased, indicating that the highly dissipative, water-swollen gel structure was being disrupted and thinned as enzymatic cleavage progressed. Although the patterns were generally similar, the kinetics over time varied between the coatings. To quantify these kinetic differences, initial rates of change immediately following enzyme introduction were calculated by fitting linear regressions to the early time data. Using the third overtone measurements, which showed better reproducibility than the fundamental frequency, we calculated degradation rates from the slopes of frequency and bandwidth versus time (Table 3).

**Table 3:** Initial Rates of Degradation Based on the Δ*f*_3_ and ΔFWHM_3_ after *P. aeruginosa* PA14^a^

film formulation	Δ*f*_3_ (Hz·min^–1^)	ΔFWHM_3_ (Hz·min^–1^)
GG100	4.58 ± 0.78	–48.22 ± 6.44
GG75:PEO25	17.85 ± 4.03	–108.3 ± 5.96

a Data presented as average ± standard deviation (*N* = 3). Statistical significance was determined using two-way ANOVA with multiple comparison and showed significance difference between films in Δ*f* (*p* = 0.01) and in ΔFWHM (*p* < 0.0001).

The rate analysis revealed significant differences between formulations. GG75:PEO25 showed frequency increase rates of 17.85 ± 4.03 Hz·min^–1^ compared to 4.58 ± 0.78 Hz·min^–1^ for GG100, an approximately 4-fold acceleration suggesting rapid bulk dissolution. Similarly, bandwidth decrease rates were −108.3 ± 5.96 Hz·min^–1^ for GG75:PEO25 versus −48.22 ± 6.44 Hz·min^–1^ for GG100. These real-time measurements provided direct confirmation that the enhanced degradation observed in bulk studies reflected genuinely faster enzymatic kinetics at the molecular level.

While the degradation responses observed with QCM-I (Figure 6) are consistent with enzymatic cleavage of the gelatin-based coatings, both bacterial and mammalian cells are known to secrete proteolytic enzymes, including metalloproteases, that could in principle interact with protein-based materials.^62^ To assess whether host-derived enzymes might contribute to similar QCM signatures, control experiments were performed using filtered supernatants from A549 epithelial cells, a widely used lung epithelial cell line that can secrete matrix-remodeling proteases under standard culture conditions.^63,64^

Introduction of mammalian cell supernatants resulted in only gradual frequency shifts and minimal changes in bandwidth (Figure S7a). Although slow mass loss or softening was observed for GG100 coatings over extended times, these responses lacked the abrupt frequency increases and pronounced ΔFWHM changes characteristic of bacterial supernatant exposure. The more hydrated GG75:PEO25 coatings exhibited even smaller responses, suggesting that host-derived protease activity under these conditions is insufficient to trigger the rapid degradation regime observed for bacterial enzymes. In the context of the presence of bacteria within a wound, the infection is not restricted to just the wound surface and in the absence of antimicrobial properties, it is highly likely to extend into the dressing further enhancing the difference in response between endogenous proteases and bacterial-derived enzymes.

To further confirm that the QCM signatures in Figure 6 arise from active bacterial proteolysis rather than other components of the bacterial supernatant, additional control experiments were performed using PA14 supernatants treated with a broad-spectrum protease inhibitor cocktail and EDTA to suppress both serine and metalloprotease activity. Under these inhibited conditions, the characteristic rapid frequency increase and bandwidth broadening were abolished, and the coatings remained largely stable following exposure to the bacterial supernatant (Figure S7b).

Together, these controls demonstrate that the pronounced QCM responses reported in Figures 6 require active bacterial proteases and are not reproduced by host-derived enzymes or by bacterial supernatants in which proteolytic activity has been suppressed.

Beyond degradation rate measurements, the relationship between frequency and bandwidth changes were analyzed to investigate the viscoelastic properties evolved during degradation. Plotting bandwidth change versus frequency change creates trajectories that reveal whether a coating is behaving more like a rigid solid, a viscoelastic gel, or a viscous liquid at each moment during the degradation process (Figure 7). These trajectories are typically interpreted relative to theoretical limits, which are highlighted in Figure 7 as a slope near zero (elastic behavior), a slope of −0.5 (Sauerbrey limit for rigid films), and steeper negative slopes approaching −2 (viscous behavior). As shown, during initial hydration in LB medium, both GG100 and GG75:PEO25 films moved toward the viscous region as water uptake caused network swelling and increased dissipation. Notably, GG100 exhibited trajectories extending well beyond the viscous limit (slopes exceeding −2), placing the response outside the range typically described by homogeneous viscoelastic film models. Such behavior has been reported for structurally nonuniform or laterally heterogeneous samples, where discrete domains or spatially varying mass distributions lead to resonance broadening that is not captured by conventional Voigt or Maxwell descriptions. Recent theoretical work using frequency-domain lattice Boltzmann modeling has shown that structured or nonuniform samples can produce disproportionately large bandwidth changes relative to frequency shifts due to heterogeneous acoustic coupling across the sensor surface.^65^

**Figure 7:**
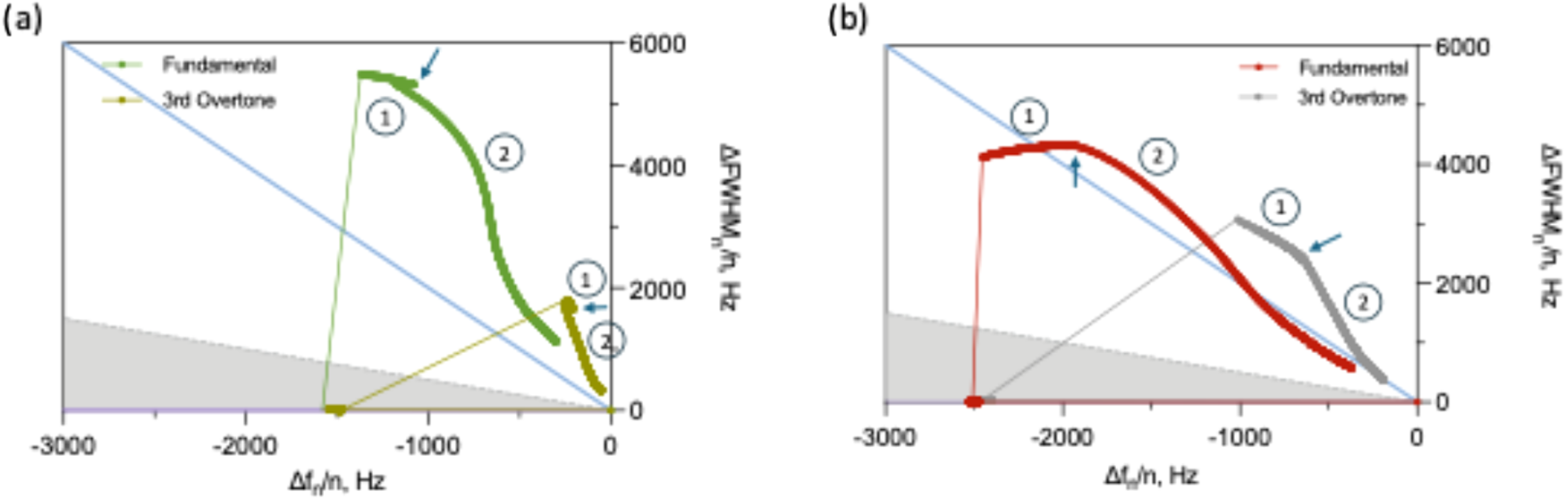
ΔFWHM vs Δ*f* plots for (a) GG100 and (b) GG75:PEO25 coatings after PA14 enzymatic exposure, using both fundamental and third overtone measurements. Theoretical “Sauerbrey”, “viscous”, and “elastic” limit lines were added as dashed gray, solid blue, and solid purple lines, respectively. Bold lines indicate data points, while finer lines were used to connect the “air” and “liquid” states. Dry (1) and hydrated (2) steps are indicated using numbers on the plots. Blue arrows indicate the time when PA14 enzymatic solution was added.

These trends are therefore interpreted as evolving structural heterogeneity at the elevated temperature, in which un-cross-linked gelatin chains tend toward liquid-like behavior while cross-linked regions remain constrained, creating nonuniform domains with different mechanical properties that oscillate out of phase with the sensor surface, resulting in anomalously high bandwidth-to-frequency ratios. Complementary atomic force microscopy-based nanoindentation measurements could be tested in future work to directly visualize these mechanical heterogeneities by mapping local stiffness across the film surface at different degradation stages.

In contrast, GG75:PEO25 remained closer to the viscous limit throughout hydration. Although this film likely exhibited comparable or greater heterogeneity due to its thicker, more swollen structure, the extensive swelling likely reduced the acoustically coupled mass to a thinner, more uniform layer at the sensor interface, leading to a behavior closer to a homogeneous viscous film.

Upon enzymatic exposure, both coatings displayed trajectories moving directly toward the origin (0,0 point), indicating progressive dissolution where both mass and dissipation decreased as degraded material detached from the surface. The GG100 trajectory showed more dramatic excursions before returning to baseline, suggesting catastrophic network failure where many peptide bonds were cleaved but fragments remained temporarily trapped within the intact siloxane scaffold, creating a mechanically weak structure that dissipated substantial energy during oscillation. GG75:PEO25 showed more direct progression toward the origin, reflecting controlled degradation where fragments escaped continuously rather than accumulating. Analysis of the trajectory slopes during enzymatic degradation, presented in Figure S8, quantitatively confirmed these distinct degradation pathways and their characteristic kinetic signatures.

Importantly, the fundamental frequency showed larger bandwidth changes than the third overtone for both coatings, consistent with depth-dependent degradation where surface-proximal regions sensed by higher overtones experienced somewhat different mechanical evolution than bulk regions probed by the fundamental resonance.^100^

To further elucidate the temporal evolution of viscoelastic properties and coupled mass during degradation, we analyzed the bandwidth-to-frequency ratio (ΔFWHM/Δ*f*) and Sauerbrey-calculated mass changes over time (Figure 8). The ΔFWHM/Δ*f* ratio represents the slope of viscoelastic trajectories in Figure 7, providing continuous monitoring of mechanical state changes through each experimental phase.

**Figure 8:**
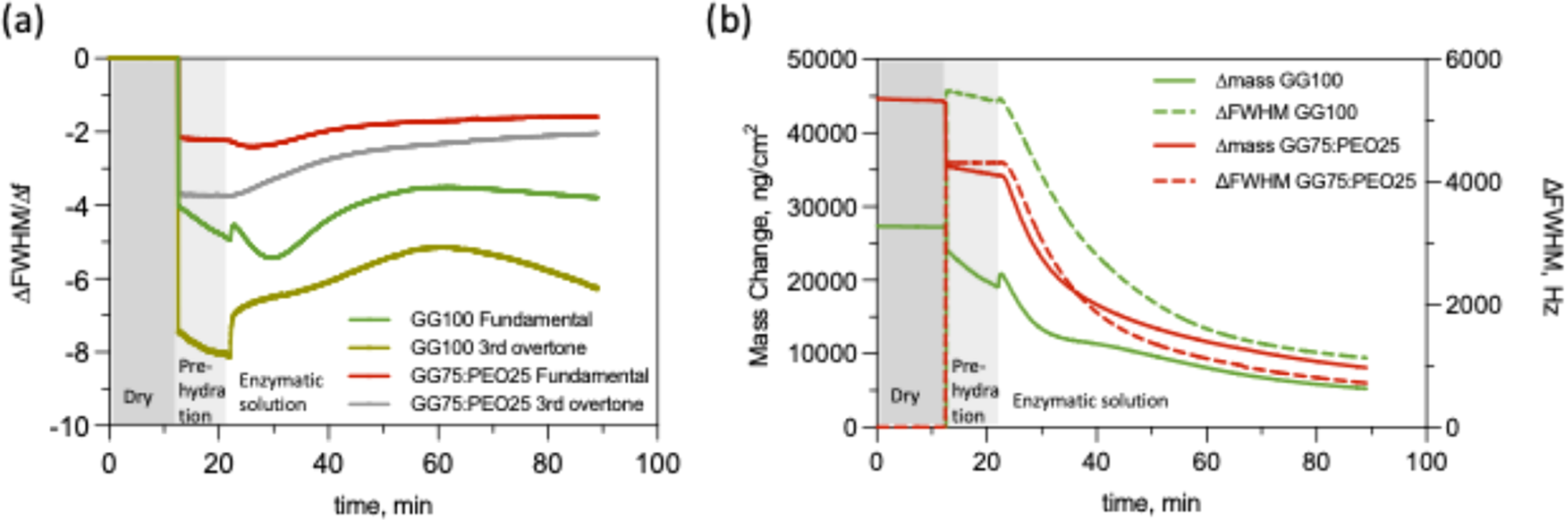
(a) ΔFWHM/Δ*f* plots over corresponding to Fundamental and 3rd overtones for GG100 and GG75:PEO25 coatings. (b) Coupled mass changes over time for both coatings calculated from Fundamental frequency changes using Sauerbrey equation. Bandwidth changes are shown in dashed lines and indicated on the right axis. The blue dotted lines separate different steps of (1) dry coated, (2) hydration with blank medium, (3) enzyme solution injection in (a) and (b). Mass changes represent baseline-corrected values using the dry and hydrated values before coating.

As shown in Figure 8a, overtone-dependent behaviors were observed particularly for GG100. Following hydration in LB medium at 37 °C, the third overtone reached ratios as high as −8 while the fundamental remained near −5. This divergence likely stemmed from depth-dependent, and/or lateral mechanical heterogeneity within the hydrated film, where the third overtone with shorter acoustic penetration depth and 9× lower lateral shear detected pronounced differences between highly swollen liquid-like domains and rigid cross-linked regions, whereas the fundamental resonance averages over a larger volume of the film. Upon PA14 enzymatic introduction, the ratio for the fundamental frequency initially became more negative indicating an increase in heterogeneity of the film as probed by the fundamental frequency. This would indicate that the less rigid portion of the films are the first to degrade and may compare with the opening of pores observed using SEM on the thicker film. However, after about 10 min of enzyme addition, both ratios decreased toward the viscous limit (−2), suggesting enzymatic activity immediately altered the structure by rapidly degrading the most accessible regions, followed by the attacking and reducing the rigidity of the rest of the film. As degradation progressed, ratios continued toward −2 and eventually reached plateau as material was progressively removed, leaving a few of the more cross-linked portions of the film.

For GG75:PEO25 films, the ratio of bandwidth-to-frequency change showed smaller variations and lower maximum values, reflecting the initial film more closely resembled a purely viscous layer on the sensor surface and indicating that each unit of mass loss was accompanied by less severe mechanical disruption. This can be interpreted as faster detachment of fragments with lower viscosity in the PEO-containing system, consistent with our proposed mechanism where PEO creates a more permeable network that facilitates fragment escape. Additionally, upon enzymatic exposure, both overtone ratios moved more smoothly toward −2, indicating a more controlled, uniform degradation.

Sauerbrey-calculated coupled mass changes provided complementary information (Figure 8b). These values were baseline-corrected, to remove the effect of water addition on the coupled mass values. Dry film deposition yielded approximately 27,000 ng/cm^2^ for GG100 and 44,000 ng/cm^2^ for GG75:PEO25, corresponding to ∼202 nm and ∼344 nm thicknesses based on the measured film densities. Notably, GG75:PEO25 produced thicker films likely due to the higher viscosity of PEO-containing solutions during spin-coating. Upon hydration in LB medium, both coatings exhibited decreases in coupled mass as GG100 coupled mass decreased to approximately 20,000 ng/cm^2^, while GG75:PEO25 decreased to ∼33,000 ng/cm^2^. This behavior suggested the transition from rigid dry films where all deposited mass contributed to the Sauerbrey signal, to swollen hydrated films where extensive water incorporation created viscoelastic layers that oscillated less rigidly with the crystal surface. The simultaneous large increases in bandwidth observed during hydration confirmed this interpretation, as the high dissipation values indicate substantial viscoelastic coupling rather than rigid behavior. The larger absolute coupled mass decrease observed for GG75:PEO25 was consistent with its more extensive swelling and greater viscoelastic character compared to GG100.

Following enzymatic solution injection, both coatings showed similar rapid mass decrease profiles, declining sharply toward baseline values. As the films softened and degraded, they progressively decoupled from the sensor, causing coupled mass to decrease rapidly. However, as shown in Table 3, initial degradation rates varied significantly, indicating enhanced hydration kinetics and enzyme accessibility in GG75:PEO25 films.

The QCM-I analysis provided several critical insights that complement and extend the end-point characterization. Time-resolved measurements demonstrated that QCM-I captures enzyme-responsive degradation through multiple complementary signatures. The frequency change rates provided quantitative kinetic information for degradation rates over time, the viscoelastic ratio evolution revealed distinct mechanical pathways for enzymatic penetration, and the coupled mass profiles confirmed the progression toward complete film removal. Finally, the multistage trajectories visible in the bandwidth-frequency plots provided direct evidence for the two-stage mechanism proposed earlier, where enzymatic cleavage creates fragments, followed by fragment removal that produce measurable mass loss. The fact that GG75:PEO25 progresses through these stages approximately four times faster than GG100, provides strong validation of our mechanistic model and confirms that network composition controls degradation kinetics across time scales.

## 4 Conclusions

This work establishes enzyme-responsive gelatin-PEO hybrid films as a viable platform for detecting bacterial infections through proteolytic activity, addressing critical limitations in current wound diagnostics. Through systematic investigation spanning molecular to macroscopic scales, we demonstrated that engineered biopolymer networks transduce bacterial enzymatic activity into measurable physical signals including mass loss, mechanical softening, and structural transformation.

The key finding is that film degradation kinetics can be precisely controlled through compositional tuning. Increasing GPTMS cross-link density produces more rigid networks with slower enzymatic degradation and brittle failure characteristics, while PEO incorporation creates hydrophilic domains that enhance water uptake and enzyme accessibility. Real-time QCM measurements revealed these compositional differences as distinct viscoelastic signatures during degradation. GG100 films exhibited slow frequency recovery and large bandwidth shifts consistent with compact network rupture, whereas hydrated GG75:PEO25 films displayed faster, smoother frequency increases characteristic of controlled surface erosion. This tunability provides a straightforward strategy for matching sensor response times to clinical requirements, where rapid-degrading high-PEO formulations could enable early detection in acute wounds while slower-degrading compositions offer sustained monitoring for chronic wound surveillance.

In this work, further validation studies are required to accelerate clinical translation. The QCM-I experiments presented here primarily employed cell-free bacterial supernatants to isolate and resolve enzymatic degradation mechanisms under controlled conditions. Importantly, complementary control experiments using mammalian cell-conditioned media were performed to assess the influence of host-derived enzymes. Nevertheless, clinical wound environments present substantially greater complexity, including intact biofilms with spatially heterogeneous enzyme production, mixed host and bacterial protease activity, and variable pH and ionic strength in wound exudates. Future work will therefore investigate film degradation in the presence of intact biofilms and clinically relevant wound mimicking fluids to further evaluate material performance under physiologically relevant conditions.

Translation to practical wound monitoring requires integration with clinically appropriate transduction mechanisms. While QCM provides valuable analytical insights into degradation kinetics and viscoelastic transitions, point-of-care applications demand simpler readout strategies. Therefore, future work will involve incorporating protease-responsive coatings derived from this system into multilayer wound dressings, or electrode-based interfaces, where bacterial enzymatic activity would induce controlled degradation. In addition, degradation-associated changes of these enzyme-responsive biopolymer coatings could be tracked and translated into practical wound monitoring devices using impedance sensors that monitor conductivity changes as films thin and dissolve.

While the present study provides the quantitative foundation required for developing activity-based infection sensors that respond to total proteolytic activity across diverse wound pathogens, translation of this platform into a deployable wound dressing will require additional engineering considerations. These include mechanical robustness under bending and stretching, compatibility with standard sterilization methods, control over functional lifetime on the wound bed, and integration into clinically relevant dressing architectures. Future studies will therefore focus on integrating the responsive films into multilayer dressing formats, assessing durability under mechanical deformation, and evaluating performance following sterilization, alongside in vivo validation.

## Supplementary Material



## Data Availability

Data will be made available on request.

## Notes

FTIR Amide I intensity changes in cross-linked gelatin films after *P. aeruginosa* exposure (Table S1); gelatin degradation assay showing bacterial protease activity (Figure S1); swelling response of gelatin films after bacterial exposure (Figure S2); visual degradation of GG:PEO films following bacterial incubation (Figure S3); SEM image of GG75:PEO25 film microstructure before exposure (Figure S4); *P. aeruginosa* attachment and penetration in GG100 and GG75:PEO25 films (Figure S5); baseline QCM-I behavior of gelatin, GG100, and GG75:PEO25 coatings in air and liquid (Figure S6); QCM-I control experiments with mammalian and protease-inhibited bacterial supernatants (Figure S7); QCM-I ΔFWHM-Δ*f* analysis of enzymatic film degradation (Figure S8) (PDF)
