# Supplementary material for: Microbial-Responsive Wound Dressings Based on Biopolymer Degradation Strategy for Detecting Bacterial Infections
Source: ACS Appl Mater Interfaces. 2026 Jan 27;18(5):7884–99. doi: 10.1021/acsami.5c22357 (PMC12903060; doi:10.1021/acsami.5c22357)
Supplement: Supplementary file 1 [file am5c22357_si_001.pdf]

Supporting Information

Microbial-responsive wound dressings based on biopolymer degradation strategy for detecting bacterial infections

Sara Sadati [a,b], Marcus J. Swann [c], Steven L. Percival [c], Jerome Charmet [a,d,e], Meera Unnikrishnan [a], Dmitry Isakov [b*]

[a] Division of Biomedical Sciences, Warwick Medical School, University of Warwick, Coventry, CV4 7AL, United Kingdom

[b] WMG, University of Warwick, Coventry, CV4 7AL, United Kingdom

[c] 5D Health Protection Group, Accelerator Building, 1 Daulby Street, Liverpool, L7 8XZ, United Kingdom

[d] School of Biomedical and Precision Engineering, University of Bern, Bern, 3008, Switzerland

[e] School of Engineering HE-Arc Ingénierie, HES-SO University of Applied Sciences and Art of Western Switzerland, 2000 Neuchâtel, Switzerland

[*] Corresponding author: d.isakov@warwick.ac.uk

Table S1. Amide I peak intensity reductions (%) in GG films after *P. aeruginosa* PA14 exposure for varying times. Values are presented as percentages. Positive values indicate intensity increases. Measurements for GG3:10 films at 6 h were not conducted.

Time (h)	GG1:10	GG2:10	GG3:10
**6**	14.41	12.88	–
**12**	–16.02	–32.42	1.38
**24**	–100.00	–24.39	18.13
**48**	–100.00	–100.00	–43.93

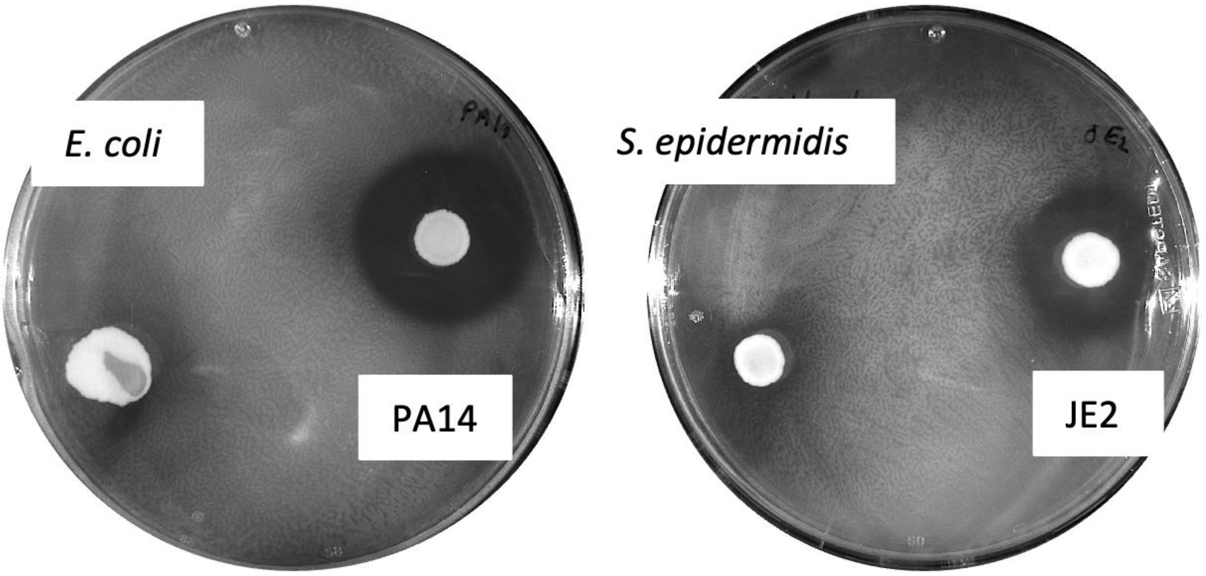

Figure S1. Qualitative assessment of bacterial protease activity using 3%w/v gelatin-supplemented agar plates incubated for 24 h at 37 ˚C with MRSA, *P. aeruginosa*, *S. epidermidis*, and *E. coli* strains. Clear zones of gelatin degradation were observed around *P. aeruginosa* and MRSA and colonies. *S. epidermidis*, and *E. coli* were included as non-proteolytic control strains and showed no clearance zones under identical conditions.

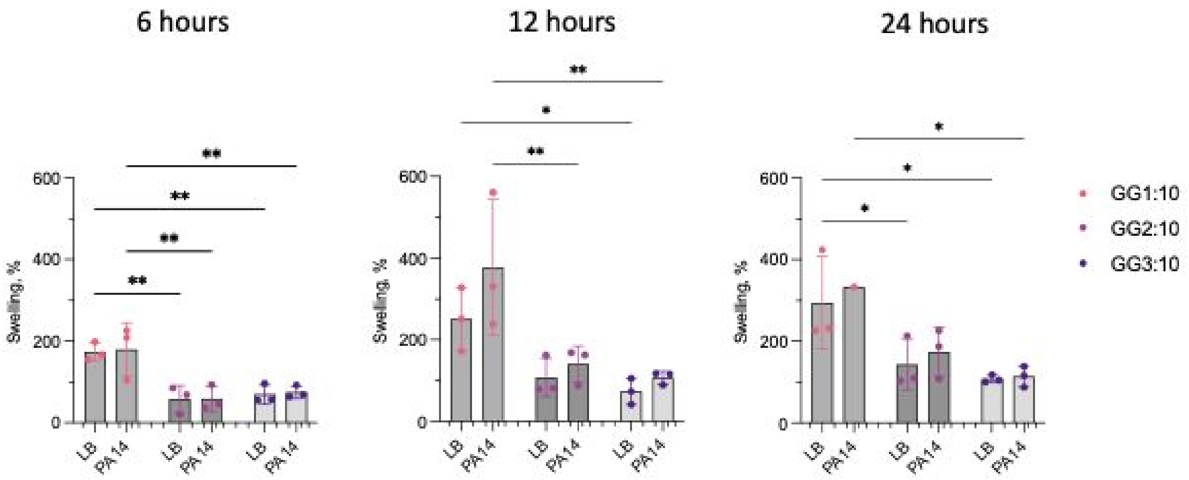

Figure S2. Swelling ratios (%) of gelatin films crosslinked with different GPTMS densities (GG1:10, GG2:10, GG3:10) after exposure to *P. aeruginosa* PA14 compared to sterile LB controls for 6, 12, and 24 hours. Data presented as mean ± standard deviation (n = 3 biological replicates). Statistical significance determined by two-way ANOVA with multiple comparisons: *p < 0.05, **p < 0.01, ***p < 0.001, ****p < 0.0001.

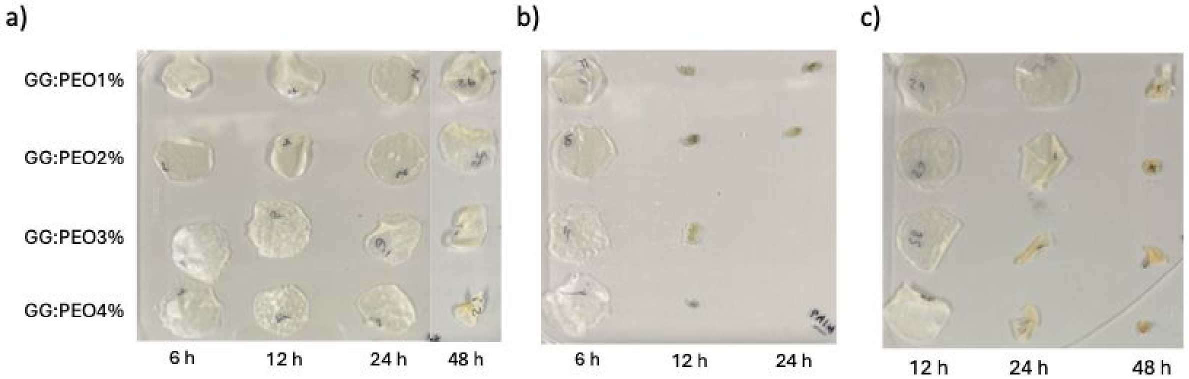

Figure S3. Visual assessment of film degradation for GG:PEO hybrid films after being exposed to (a) sterile control (b) *P. aeruginosa*, and (c) MRSA strains over 48 h.

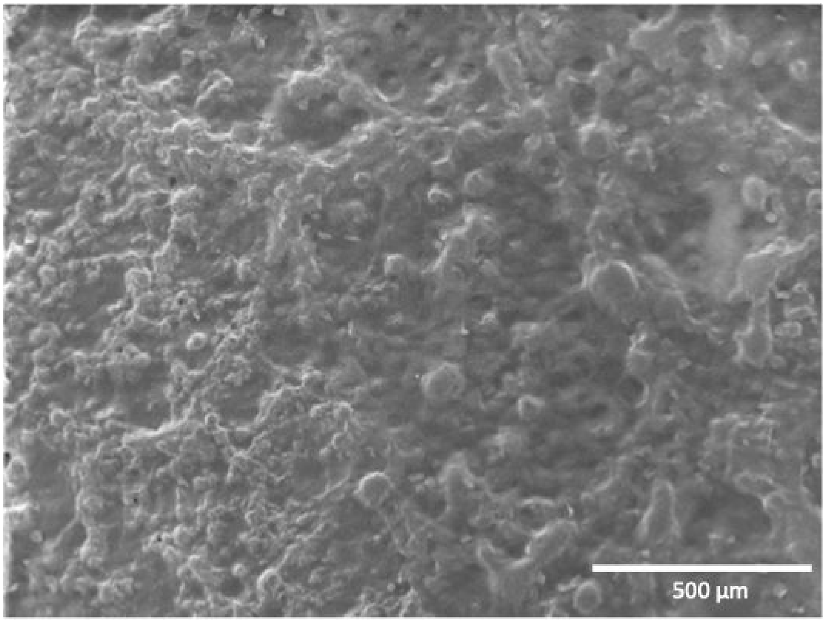

Figure S4. Representative SEM micrograph of GG75:PEO25 films at 0 h time point before exposure to blank controls or bacteria cultures. Scale bar indicates 500 µm.

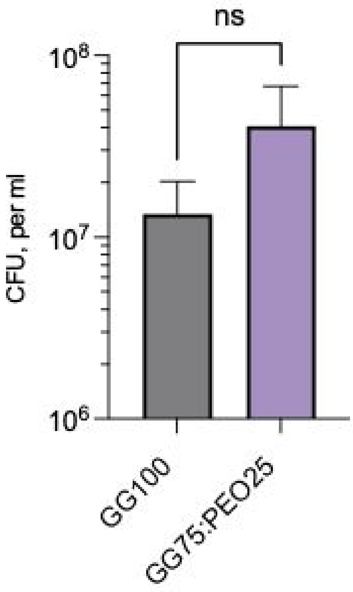

Figure S5. Colony-forming unit (CFU) counts for PA14 cells attached/penetrated in GG100 and GG75:PEO25 films following 12 h incubation. Unpaired Student's t-test revealed no statistical significance between the films. Data presented as mean ± standard deviation (N = 3 biological replicates).

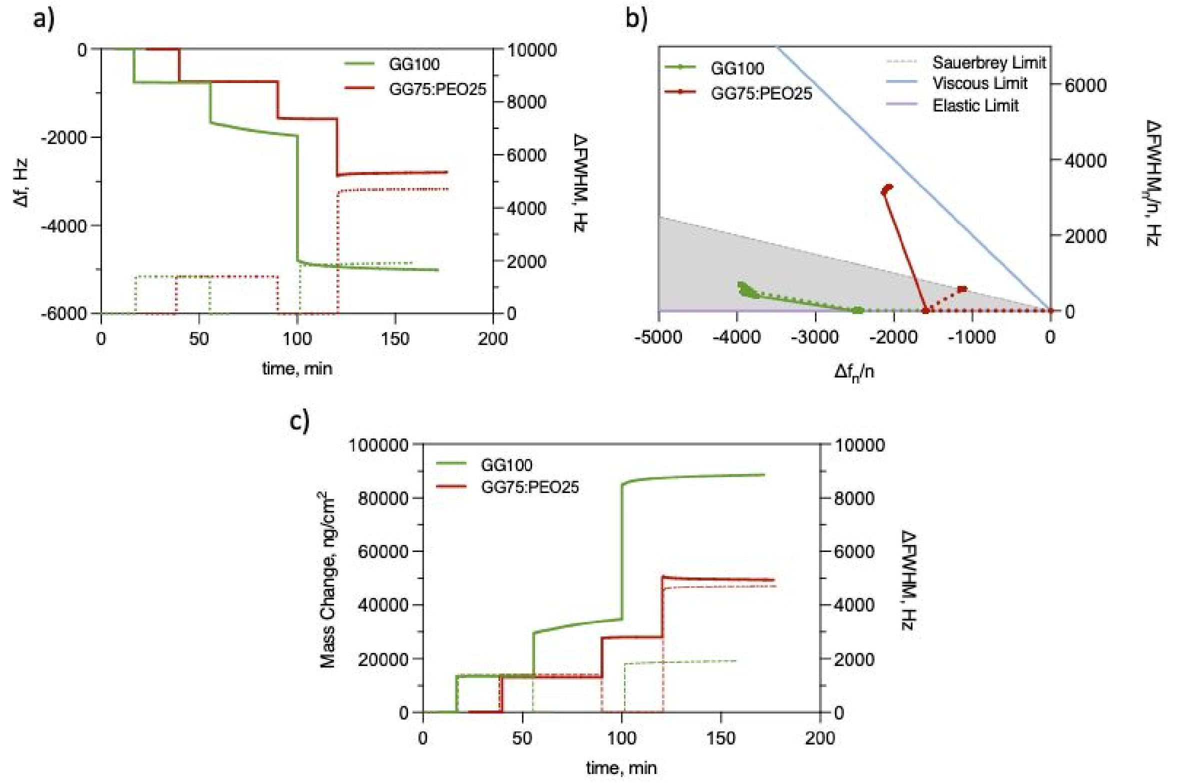

Figure S6. Baseline behavior of GG100 and GG75:PEO25 coatings under air and aqueous conditions. (a) Δf and ΔFWHM plots over time comparing the frequency and bandwidth changes of the coatings in air and liquid, using fundamental overtone measurements. Left axis and solid lines show the resonance frequency changes, right axis and dashed lines present bandwidth changes over time. Sequence of steps for bare sensors coated in: air, water, dry coating in air, coating in water (b) ΔFWHM vs. Δf plots comparing the viscoelastic behavior of the coatings in air and liquid, using both fundamental (solid lines) and 3rd overtone (dashed lines) measurements. Theoretical "Sauerbrey", "viscous", and "elastic" limit lines were added for better visualization. Bold lines indicate data points, while finer lines were used to connect the "air" and "liquid" states. (c) Sauerbrey mass changes and dissipation changes through ΔFWHM for GG100, and GG75:PEO25 coatings. Graphs were shifted horizontally by cropping baseline values for better representation without overlaps. Measurements were taken at room temperature. Sequence of steps for bare sensor coated in: air, water, dry coating in air, coating in water. Measurements were taken at the room temperature.

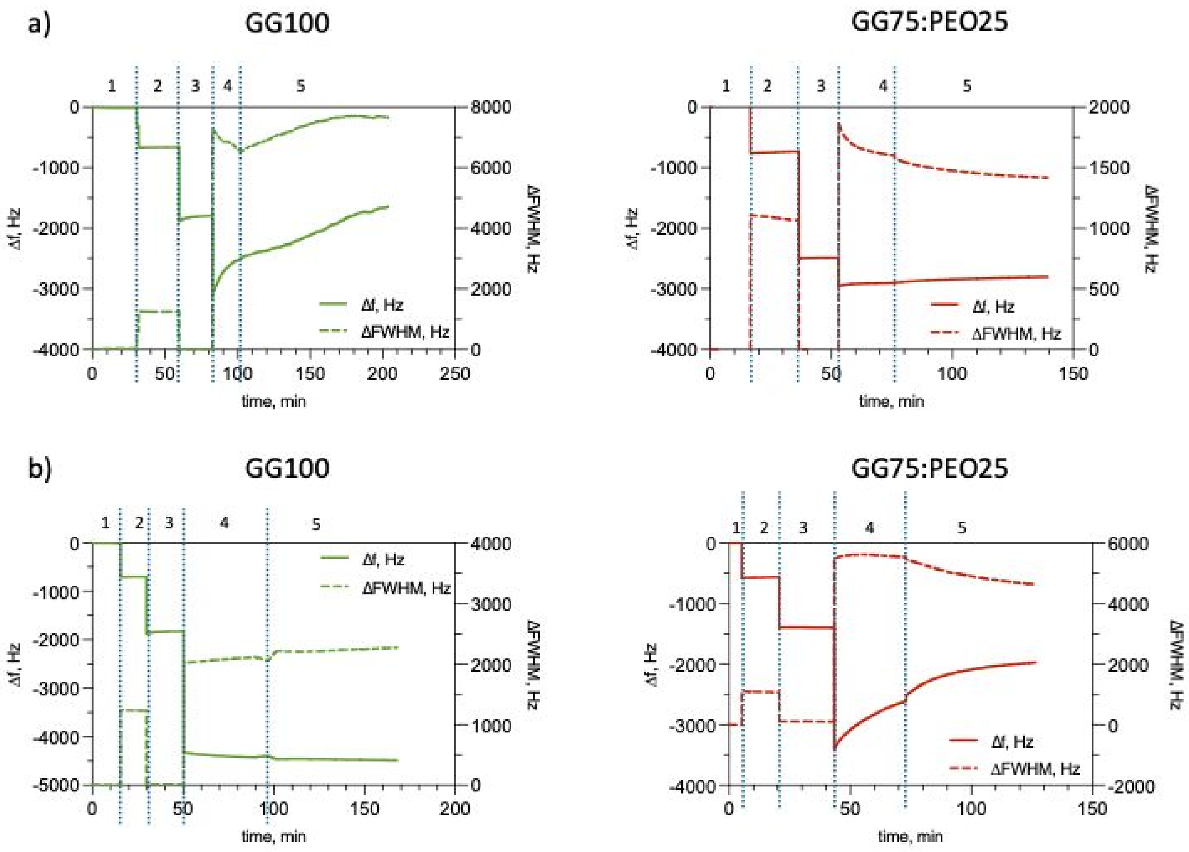

Figure S7. QCM-I control data using mammalian cell supernatants and bacterial supernatants treated with protease inhibitors. Traces show changes in resonance frequency (Δf) and bandwidth (ΔFWHM) for GG100 and GG75:PEO25 coatings. Numbers indicate experimental steps: (1) uncoated dry baseline, (2) uncoated wet baseline, (3) dry coated state, (4) blank medium pre-hydration (cell culture medium or LB), and (5) exposure to either filtered A549 epithelial cell supernatant (a) or protease-inhibitor treated PA14 supernatant (b).

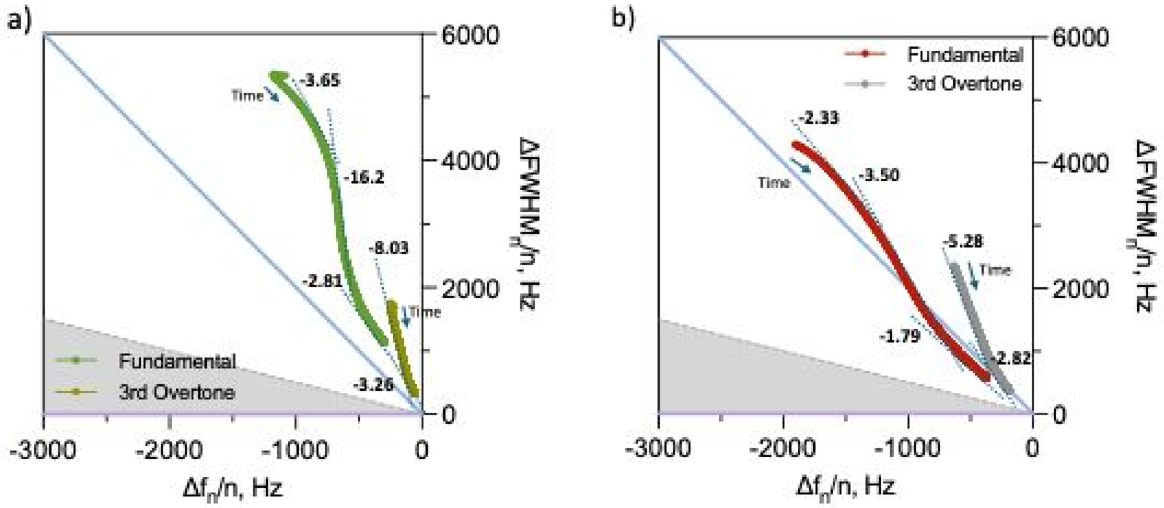

Figure S8. ΔFWHM vs. Δf plots corresponding to Figure 7 after the injection of PA14 enzymatic solution for (a) GG100 and (b) GG75:PEO25 coatings. The diagonal blue dotted lines indicate different phases of film degradation, characterized by a different ΔFWHM to Δf ratio (shown by the numbers next to the lines). This analysis was done using linear regression for both fundamental and 3rd overtones. For both coatings, the 3rd overtone showed a two-step transition, while the fundamental frequency exhibited a more complex three-step behavior.
